# Text-to-Korean Sign Language Pose Sequence Generation Using Non-Manual Signal Conditioning and Multi-Scale Temporal Refinement

**DOI:** 10.3390/s26134245

**Published:** 2026-07-04

**Authors:** Seungju Lee, Gooman Park

**Affiliations:** Department of Smart ICT Convergence Engineering, Seoul National University of Science and Technology, 232 Gongneung-ro, Nowon-gu, Seoul 01811, Republic of Korea; sjleee05@gmail.com

**Keywords:** Korean sign language, sign language generation, text-to-pose generation, non-manual signals, multi-scale temporal refinement, length prediction, accessibility

## Abstract

Automatic sign language generation has the potential to support information accessibility for deaf and hard-of-hearing individuals. Generating sign language pose sequences from natural language text can serve as an intermediate representation for avatar-based sign language expression and sign language video synthesis. However, text-to-sign pose generation is challenging because sign language conveys meaning through both manual movements and non-manual signals, while requiring temporally coherent motion over local and sentence-level contexts. In addition, text length does not directly correspond to the number of pose frames required for sign language expression. To address these issues, this study proposes a text-to-Korean Sign Language (KSL) pose generation model based on non-manual signal conditioning and multi-scale temporal refinement. The proposed framework integrates a text encoder, pose decoder, non-manual signal conditioning, multi-scale temporal refinement, and length prediction/blending. The model generates normalized 58-joint KSL keypoint sequences from morpheme-level text inputs and jointly optimizes pose reconstruction, motion continuity, bone consistency, PCK-aware precision, non-manual signal prediction, and length consistency. Experimental results on a KSL text–pose dataset show that the proposed model outperforms text-only and Transformer-based baselines. Compared with the Transformer text-to-pose baseline, the proposed model reduced MPJPE from 0.408236 to 0.316366 and Pose MAE from 0.165473 to 0.128570. It also improved PCK@0.05 from 0.136090 to 0.163928 and reduced the length relative error from 0.221455 to 0.127152. In particular, the best-threshold non-manual F1 substantially increased from 0.010859 to 0.494566. These results suggest that text-based KSL pose generation should jointly consider non-manual expressions, length consistency, and long-term temporal motion structure rather than relying only on frame-wise keypoint prediction. However, the reported improvements should be interpreted as coordinate- and label-level evidence, not as a complete validation of linguistic meaningfulness or real-world accessibility.

## 1. Introduction

### 1.1. Research Background

Sign language is a primary means of communication for deaf and hard-of-hearing individuals. It is a visual language that conveys meaning through various bodily expressions, including not only hand and arm movements but also facial expressions, eye gaze, head movements, and upper-body orientation [1,2,3,4]. With recent advances in artificial intelligence-based natural language processing and motion generation technologies, the demand for sign language-based information delivery has increased beyond conventional speech- and text-centered accessibility [5,6]. In particular, automatic conversion of textual information into sign language expressions can serve as an important assistive technology in public services, education, broadcasting, guidance systems, healthcare, and disaster information delivery [5,6,7].

Automatic sign language generation aims to convert an input natural language sentence into a sign language video or a sign language pose sequence [8,9,10]. Among these approaches, pose sequence generation has several advantages over direct video generation, including relatively lower data requirements, lower computational cost, and easier quantitative evaluation of generated results [8,10,11]. In addition, pose sequences can be used as intermediate representations for avatar-based sign language expression, three-dimensional character animation, and sign language video synthesis models [9,12,13]. Therefore, generating sign language pose sequences from text can be regarded as a key step in automatic sign language generation [8,9,14].

Korean Sign Language (KSL) has an independent grammatical system that differs from spoken Korean and shares the general linguistic properties of sign languages, including spatial structure, directionality, and non-manual signals [1,3,15]. Existing Korean sign language resources and datasets have supported recognition and translation research, but text-based KSL pose generation remains relatively underexplored [16,17,18]. Therefore, converting Korean text into KSL pose sequences is more complex than a general text-to-motion generation problem [19,20,21]. In particular, in KSL, meanings that cannot be sufficiently expressed by hand movements alone are often conveyed through non-manual signals such as facial expressions, head movements, and upper-body posture [3,22,23]. Considering these characteristics, an automatic KSL generation model should not merely generate hand and arm coordinates corresponding to sentence-level semantics, but should also reflect the linguistic characteristics and temporal motion structure of sign language [8,10,11].

### 1.2. Limitations of Previous Studies

Previous studies on text-based sign language generation and pose generation have mainly focused on generating pose sequences centered on the hands, arms, and torso from input sentences [8,9,10,24]. Although such approaches are effective for modeling manual movements, they have limitations because meaning in sign language is not conveyed solely through hand movements [3,22,23]. In actual sign language communication, non-manual signals such as facial expressions, mouth shapes, eye gaze, head nods, and torso tilts play important roles in expressing interrogative forms, negation, emphasis, emotion, and pragmatic meaning. Therefore, generation models that focus primarily on manual movements may not sufficiently ensure the linguistic completeness and naturalness of sign language expressions.

In addition, non-manual signals are often not explicitly treated as generation conditions in previous pose-based sign language production studies [8,10,24]. Some models include facial or upper-body joints as part of the overall pose coordinates, but they do not interpret them as separate semantic signals or condition the generation process on them [9,10,13]. In such cases, the model learns non-manual signals merely as coordinate regression targets, making it difficult to sufficiently capture the relationship between textual meaning and non-manual expressions. This limitation can lead to degraded generation quality, especially in languages such as KSL, where non-manual signals are crucial for semantic distinction.

Temporal consistency is another important issue. Sign language is not composed of static poses in individual frames, but of continuous movements that evolve over time [10,15,23]. Similar temporal consistency issues have also been discussed in general text-to-motion and human motion generation studies [19,20,21,25]. However, many pose generation models focus on reducing frame-wise coordinate errors, and thus may fail to sufficiently preserve the overall motion flow or long-term temporal context of the sequence. As a result, although the generated poses may appear plausible in individual frames, discontinuities or unnatural jitter may occur when the sequence is played continuously. In particular, as sentence length increases, it becomes more difficult to stably generate connectivity between preceding and following motions, motion transitions, repetitive structures, and ending motions.

Finally, there exists a length mismatch problem between text and sign language pose sequences. The length of a natural language sentence does not correspond one-to-one with the number of frames required for its sign language expression [8,10,14]. Similar alignment and duration issues have also been noted in text-conditioned human motion generation, where linguistic length and motion duration are not directly matched [19,25,26]. A short sentence may require a long motion sequence in sign language, whereas a long sentence may be expressed in a compressed manner. Therefore, fixed-length generation or simple length interpolation can cause mismatches between sentence meaning and motion duration. This length mismatch affects not only pose accuracy but also motion speed, expression duration, and the overall naturalness of the generated sequence.

### 1.3. Research Objectives

The objective of this study is to develop and evaluate a technical framework for generating Korean Sign Language (KSL) pose sequences from natural language text inputs. Rather than claiming to produce deployable sign-language content, this study focuses on text-to-pose generation as an intermediate step toward future KSL avatar or video generation systems. To this end, the model generates normalized KSL keypoint sequences from textual semantic representations and incorporates non-manual signal conditioning and multi-scale temporal refinement to better reflect important structural properties of KSL. The proposed model is therefore designed to improve frame-wise coordinate prediction, temporal continuity, and non-manual expression modeling, while recognizing that these coordinate-level improvements do not by themselves guarantee full linguistic meaningfulness or real-world accessibility.

First, this study addresses the problem of text-to-KSL pose generation, in which KSL pose sequences are generated from text inputs. An input sentence is converted into a semantic representation through a text encoder, and a pose generation decoder generates a sign language pose sequence conditioned on this representation. The generation target includes not only manual movements centered on the hands and arms, but also pose information related to non-manual expressions, including the face, head, and upper body.

Second, this study explicitly conditions the pose generation process on non-manual signals. Because non-manual signals play an important role in conveying meaning and improving the naturalness of sign language expressions, they are not treated simply as additional coordinates, but are used as part of the generation conditions. This design encourages the model to learn associations between textual meaning and non-manual expressions. However, whether a generated sequence is fully recognizable as natural KSL cannot be verified by coordinate-based metrics alone, and must ultimately be assessed through KSL expert or user evaluation.

Third, this study improves the temporal quality of generated poses through a multi-scale temporal refinement structure. Sign language motions include both local movements over short intervals and long-term motion flow across the entire sentence. Accordingly, this study applies a temporal refinement structure that refines pose sequences at multiple temporal scales and captures long-term temporal context. In addition, the model jointly optimizes pose accuracy and motion naturalness by considering not only coordinate errors, but also velocity, acceleration, jerk, bone consistency, and PCK-aware objectives.

### 1.4. Main Contributions

The main contributions of this study are as follows.

First, we propose a text-to-KSL pose generation model based on non-manual signal conditioning. The proposed framework explicitly considers non-manual signals, which are important for meaning delivery in KSL. Unlike previous pose generation methods that mainly focus on manual movements, the proposed method conditions the generation process on non-manual information related to the face, head, and upper body, thereby improving the modeled correspondence between textual representations and pose-level non-manual expression cues.

Second, we introduce a temporal refinement structure based on multiple temporal scales. A sign language pose sequence includes not only local hand movements but also the temporal flow of the entire sentence. The proposed method models both short-term movements and long-term temporal context through multi-scale temporal refinement. This improves the temporal continuity of generated poses and the naturalness of motion transitions.

Third, we apply a training strategy that considers motion naturalness and length consistency. Instead of relying solely on coordinate reconstruction loss, this study jointly optimizes dynamic motion quality by combining velocity, acceleration, jerk, bone consistency, and PCK-aware losses. In addition, length prediction and length blending strategies are applied to alleviate the mismatch between text length and sign language pose sequence length.

Fourth, we validate the effectiveness of the proposed method through component-wise ablation studies. This study analyzes the effects of non-manual signal conditioning, multi-scale temporal refinement, motion losses, PCK-aware objectives, length prediction, and refinement modules. Through these analyses, we quantitatively examine how each component and their combined effects contribute to KSL pose generation performance.

In summary, this study proposes a text-based KSL pose sequence generation framework that jointly models non-manual signals and sentence-level temporal context. The proposed method emphasizes that sign language generation models should be evaluated not only through joint coordinate prediction, but also with respect to length consistency, motion stability, non-manual expression behavior, and eventually linguistic meaningfulness. The framework should therefore be interpreted as a technical intermediate representation model for future avatar-based KSL generation and sign language video synthesis, rather than as a complete accessibility system ready for real-world deployment.

## 2. Related Work

### 2.1. Sign Language Translation and Sign Language Generation

Artificial intelligence research on sign language can be broadly categorized into sign language recognition, sign language translation, and sign language production [5,6,27]. Sign language recognition aims to predict sign words, glosses, or sentences from videos or pose sequences, whereas sign language translation includes the conversion of sign language expressions into natural language sentences or the conversion of natural language sentences into sign language expressions [28,29]. In contrast, sign language generation aims to generate sign language poses, avatar motions, or sign language videos from input natural language, glosses, or semantic representations [8,9,10]. In particular, automatic sign language generation has received increasing attention as a key technology for improving information accessibility for deaf and hard-of-hearing individuals [5,6,7].

Early studies on sign language generation mainly relied on rule-based avatars or gloss-based animation systems [5,6]. These approaches can generate relatively stable results by combining predefined sign words and motion templates. However, they have limitations in representing diverse sentence structures and natural motion variations [6,9]. With the introduction of neural network-based methods, studies on generating sign language poses or videos from natural language sentences have been actively conducted [8,9,10,24,30]. Representative Text2Sign-type studies proposed a staged approach in which a natural language sentence is first converted into a sign language pose sequence, and a sign language video is then generated based on the pose sequence [9]. Although these studies are important in demonstrating the feasibility of neural sign language generation, natural sign language expression requires not only pose accuracy but also motion continuity, expression length, and facial and upper-body expressions [3,10,22,23].

Recently, there has been increasing interest in directly generating sign language poses or videos from text without using glosses as intermediate representations [13,14,31,32,33]. Such end-to-end approaches have the advantage of reducing dependence on gloss annotations. However, they are difficult to train because the model must directly learn the structural differences between natural language and sign language. Moreover, sign language is not a simple sequence of hand movements, but a multi-channel visual language that combines the face, head, upper body, spatial directionality, and temporal rhythm [1,3,15,22,23]. Therefore, general text-to-motion generation models cannot be directly applied to sign language generation without modification. To address these challenges, recent studies have explored three-dimensional signing avatar generation, diffusion-based sign language generation, and large language model-based sign language generation [12,13,14,31,32,33].

However, many existing studies have focused on datasets for several sign languages, such as German Sign Language, American Sign Language, and British Sign Language, while text-based pose sequence generation for KSL remains relatively limited [8,9,16,17,18,28,29]. KSL has an independent grammatical system that differs from spoken Korean and exhibits unique characteristics in sentence structure, spatial use, and non-manual signals. Therefore, automatic KSL generation requires a model design that reflects both the linguistic characteristics of KSL and the temporal properties of pose sequences, rather than simply applying the general structure of existing sign language generation models.

### 2.2. Text-Based Pose Sequence Generation

Text-based pose sequence generation is the task of generating a sequence of joint coordinates representing human motion conditioned on a natural language sentence. This field is closely related to human motion generation or text-to-motion generation, and aims to convert the meaning of an input sentence into temporally continuous motion. Previous studies have employed various generative architectures, including recurrent neural networks, sequence-to-sequence models, Transformers, variational models, and diffusion models [19,20,21,25,26,34,35,36]. Recently, text-to-motion studies that generate diverse three-dimensional human motions from natural language descriptions have been actively investigated, including language-based motion synthesis and editing approaches [19,25,36]. Diffusion-based models have also shown strong potential in modeling multiple plausible motion distributions [20,21,37].

Sign language pose generation shares a common objective with general human motion generation in that it generates temporal pose sequences [8,10,19,21]. However, sign language generation has more complex characteristics than general human motion generation because it requires the preservation of linguistic meaning, manual articulation, non-manual expression, and temporal rhythm [3,6,22,23]. In a typical text-to-motion task, the main objective is to represent action meanings such as “walking,” “jumping,” or “sitting.” In contrast, sign language generation must precisely convert the specific meaning of a natural language sentence into hand shapes, hand positions, movement directions, repetition patterns, facial expressions, and upper-body postures. In other words, sign language pose sequence generation is a challenging sequence-to-sequence problem that requires both semantic preservation and motion naturalness.

Sequence-to-sequence-based pose generation methods encode an input sentence and then generate frame-wise joint coordinates through a decoder [8,9,10,38]. These methods have the advantage of learning a direct correspondence between text and pose sequences. However, if the model focuses mainly on frame-wise coordinate prediction, the long-term motion flow and temporal consistency of the generated sequence may be weakened. In particular, in sign language, motion onset, development, transition, and termination are closely related to sentence meaning [10,15,23]. Therefore, it is difficult to generate natural sign language sequences using only simple frame-wise reconstruction loss.

The length mismatch between text and pose sequences is also an important issue in both sign language production and text-conditioned motion generation [8,10,14,26]. The number of tokens in an input sentence does not directly correspond to the number of output pose frames, and even sentences of the same length may require different numbers of frames depending on signing speed or motion complexity. Therefore, a text-based sign language pose generation model should predict not only the semantic representation of the input text, but also the appropriate output length, motion speed, and temporal alignment. Considering these issues, this study incorporates length prediction and length blending strategies and introduces a structure that refines the generated pose sequence at multiple temporal scales.

### 2.3. Modeling Sign Language Expressions Based on Non-Manual Signals

Sign language is not composed solely of hand and arm movements. Non-manual signals, such as facial expressions, mouth shapes, eyebrow movements, eye gaze, head movements, and upper-body orientation, perform grammatical and semantic functions in sign language [3,15,22,23]. For example, non-manual signals can be used to express interrogatives, negation, conditionals, emphasis, emotion, and the speaker’s attitude. In some cases, they convey semantic distinctions that are difficult to distinguish using manual movements alone. Therefore, automatic sign language generation models must explicitly consider non-manual signals to generate results that are closer to real sign language expressions.

Some existing studies on sign language generation have proposed modules for generating facial expressions or non-manual features [10,12,13]. For example, signing avatar studies have attempted to improve the naturalness of sign language expressions by separately synthesizing facial animation or non-manual signals. Recent studies have also attempted to use large language models, autoregressive generation, or diffusion-based frameworks to predict glosses, latent sign representations, or non-manual markers from input sentences and connect them to the motion generation stage [14,31,32,33]. These trends indicate that non-manual signals are not merely auxiliary elements, but essential components for meaning delivery in sign language generation.

However, in many existing pose-based sign language generation models, non-manual signals are treated only as part of the overall joint coordinates or are not used as separate conditional information [8,10,24]. In such cases, even if the model predicts coordinates related to the face, head, and upper body, it may not explicitly learn how these expressions are connected to the meaning of the input text. In particular, because non-manual signals often occur simultaneously with manual movements or are activated only during specific intervals, it is difficult to stably model their occurrence timing and duration using simple coordinate regression.

To alleviate these limitations, this study explicitly conditions the pose generation process on non-manual signals. That is, non-manual signals are not regarded simply as part of the generation output, but are used as intermediate conditions that connect textual meaning and pose sequences. This enables the model to reflect facial, head, and upper-body expressions that are important in KSL during pose generation and encourages the generation of linguistically meaningful expressive structures beyond manual movement-centered models.

### 2.4. Generation Models Based on Temporal Context and Motion Refinement

Temporal context modeling is a key factor that determines the naturalness and consistency of generated pose sequences. Human motion has a continuous temporal structure, and both smooth changes between adjacent frames and motion flow over long intervals are important. In particular, sign language is a visual language in which semantic units are temporally arranged. Therefore, the overall semantic structure of a sentence and motion transitions must be expressed stably. Accordingly, sign language pose generation models should consider both local frame-level changes and long-term temporal context.

Transformer-based models are widely used in pose generation, human motion generation, and sign language generation because they can model dependencies within long sequences through self-attention [8,10,29,35,38]. However, a standard Transformer architecture alone may not stably preserve both fine-grained local movements and long-term motion flow. To address this limitation, recent studies in human motion generation have proposed local-to-global modeling, multi-scale temporal modeling, temporal refinement, and diffusion-based refinement methods [19,20,21,25,37,39,40]. These methods have evolved toward improving naturalness and consistency by combining features from different temporal ranges or by iteratively correcting generated motion.

Diffusion-based motion generation progressively restores motion sequences from noise and has strengths in modeling diverse plausible motion distributions [20,21,41,42]. In text-conditioned motion generation, diffusion-based approaches can be effective because multiple natural motions may exist for the same sentence [20,21,37]. Similar diffusion-based ideas have also recently been applied to sign language production and sign language video generation [31,32,33,43,44]. However, diffusion models require high computational cost, and for sequences such as sign language, where semantic precision is important, not only motion realism but also textual meaning, manual motion accuracy, and synchronization of non-manual expressions should be jointly considered.

Based on these trends, this study introduces a multi-scale temporal refinement structure. The proposed model does not process generated pose sequences at a single temporal resolution. Instead, it uses multiple temporal kernels and temporal scales to refine both local movements and long-term context. In addition, velocity, acceleration, jerk, bone consistency, and PCK-aware objectives are included in the training objective to jointly optimize not only static pose coordinate accuracy but also dynamic motion naturalness. Through this design, this study alleviates the temporal instability of existing text-based pose generation models and proposes a long-term context-aware generation structure suitable for KSL pose sequences.

### 2.5. Differences from Previous Studies

Existing studies on sign language generation have provided important foundations for converting natural language or glosses into sign language poses or videos [8,9,10,13,14,24,31]. However, most studies have focused on specific sign language datasets or manual movement-centered pose generation, and have limitations in explicitly reflecting the linguistic characteristics and non-manual signals of KSL. In addition, although general text-to-motion studies are effective for generating natural human motions [19,20,21,25,35], additional structures are required for linguistic motion generation tasks such as sign language, where semantic precision and temporal synchronization are crucial [6,10,11].

Based on these limitations, this study differs from previous studies in the following aspects. First, it targets KSL pose sequence generation and generates pose sequences that include both manual and non-manual expressions from input text. Second, it uses non-manual signals not merely as part of the generation output, but as explicit conditions in the pose generation process. Third, it models both short-term movements and sentence-level long-term temporal context through multi-scale temporal refinement. Fourth, it applies a training strategy that considers motion naturalness, length consistency, and PCK-based pose quality in addition to coordinate reconstruction loss. Finally, it quantitatively verifies the effects of non-manual conditioning, temporal refinement, motion losses, and length prediction through ablation studies.

Therefore, this study can be regarded as an extension of existing sign language generation and general pose generation research to the linguistic characteristics of KSL. In particular, it is differentiated by addressing the text-to-KSL pose sequence generation problem through the combination of non-manual signal conditioning and multi-scale temporal refinement.

## 3. Dataset and Problem Definition

### 3.1. Korean Sign Language Pose Sequence Dataset

In this study, we use the AI Hub KSL video dataset as the source data for constructing text–pose pairs [45]. The dataset source page and its accompanying data description specify that the original AI Hub data provide Korean sentence or word expressions, morpheme-level annotations, video metadata, signer information, and keypoint JSON files. The dataset is suitable for this study because it includes both sentence-level and word-level KSL expressions together with body, hand, and face keypoints that can be converted into pose sequences.

**Figure 1:**
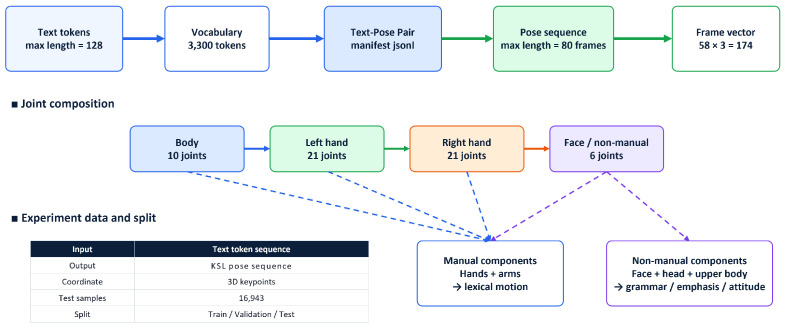
Dataset and pose representation.

Figure 1 summarizes the dataset construction and pose representation used in this study.

In the experiments, we used real-captured frontal-view samples to reduce variation caused by camera viewpoint and recording type. Specifically, the preprocessing pipeline selected real-captured samples (REAL), frontal-view samples (F), and both sentence-level (SEN) and word-level (WORD) expression types. Each sample corresponds to one KSL expression unit and contains an input text sequence, a pose sequence, non-manual signal tags, signer information, and recording-condition metadata. The use of both sentence-level and word-level samples allows the model to learn short isolated signs as well as longer sentence-level motion patterns.

The training, validation, and test sets were constructed from independent manifest files. The manifest files store the sample identifier, text token IDs, pose-file path, non-manual multi-hot label, pose length, signer metadata, and recording metadata. We used a signer-level split when signer metadata were available, so that the same signer was not intentionally shared across different splits. This split was used to reduce signer-specific memorization and to evaluate generalization to signer variation. However, because the public dataset contains an imbalanced number of samples across signers and recording subsets, the split should be interpreted as a controlled experimental split rather than a complete subject-independent benchmark over all possible signers.

In the test stage, prediction and evaluation were performed on 16,943 samples. This number denotes the number of test-set text–pose pairs after applying the preprocessing filters described above, not the total size of the original AI Hub dataset. Each of the 16,943 samples consists of one input text sequence and one corresponding KSL pose sequence with a valid pose length.

The input text consists of morpheme-level token sequences extracted from the original morpheme annotations. The textual information of each sample is converted into integer token IDs based on a vocabulary, and padding tokens are used to align the sequences within a batch. In the final experimental setting, the maximum text length was limited to 128. Inputs longer than this limit were truncated for training stability and computational efficiency, whereas shorter inputs were padded for batch processing.

The original data used in this study were not collected with wearable sensors, data gloves, or depth sensors by the authors. Instead, the pose data were derived from the keypoint annotations distributed with the AI Hub RGB video dataset. This differs from studies that directly estimate poses using general pose-estimation pipelines or depth-based three-dimensional pose-estimation models [46,47,48]. The original keypoint files include body, left-hand, right-hand, and face keypoints with three annotation channels per joint. We did not train an additional pose-estimation model for this study; rather, we converted the provided keypoint annotations into a unified 58-joint representation for text-to-pose learning.

The output is a KSL pose sequence with a temporal dimension. During preprocessing, only the major joints required for sign language generation were selected from the original keypoint annotations. The final pose representation consists of 58 joints, each represented by three normalized keypoint channels, for each frame. Therefore, the pose vector of a single frame has a dimension of 58×3=174, and the model generates a pose sequence of up to 80 sampled frames from the text input.

The pose components can be broadly divided into manual and non-manual regions. The manual region includes hand and arm movements and directly contributes to representing the shape, location, direction, and motion of sign language words. The non-manual region includes joint information related to the face, head, and upper body, and supports or distinguishes grammatical and semantic elements of sign language expressions, such as questions, negation, emphasis, emotion, and sentence type. In this study, both manual and non-manual components are generated as a single pose sequence. In addition, non-manual signals are used as separate conditional information to reflect the linguistic characteristics of sign language expressions.

### 3.2. Pose Representation

The pose sequence used in this study is represented as a tensor consisting of temporal, joint, and keypoint-channel dimensions. A pose sequence for one sample can be expressed as follows:

(1) $$Y=\left\{y_{t,j}|t=1,\ldots ,T,\;j=1,\ldots ,J\right\}\in R^{T\times J\times C},$$

where *T* denotes the length of the pose sequence, *J* denotes the number of joints, and *C* denotes the number of normalized keypoint channels per joint. In this study, we use J=58 and C=3; therefore, the pose of each frame can be flattened into a 174-dimensional vector:

(2) $$y_{t}\in R^{J\times C}=R^{58\times 3},\;\mathrm{vec}\left(y_{t}\right)\in R^{174}.$$

The joint configuration consists of body, left-hand, right-hand, and face regions. In the original keypoint format, the body contains 25 joints following the OpenPose format [46]. However, this study selects 10 upper-body-centered body joints that are relatively important for sign language expression. Each hand contains 21 joints; thus, the left and right hands include 42 hand joints in total. For the face region, six major face joints related to non-manual expressions are selected. Therefore, the final joint configuration is defined as follows:

(3) $$J=J_{\mathrm{body}}+J_{\mathrm{left}\;\mathrm{hand}}+J_{\mathrm{right}\;\mathrm{hand}}+J_{\mathrm{face}}=10+21+21+6=58.$$

The manual pose components mainly consist of movements of both hands and arms. This region represents the core shape and motion trajectory of sign language words and is therefore important for pose accuracy evaluation. In contrast, the non-manual pose components include facial keypoints, body joints related to head movements, and body joints reflecting upper-body orientation. These regions are necessary for representing sentence type, emphasis, emotion, and pragmatic meaning that cannot be expressed by hand movements alone.

The pose channels were obtained from the AI Hub keypoint annotation fields rather than from a sensor stream. The original annotation fields were converted into the unified joint order used in this study: upper-body body joints, 21 left-hand joints, 21 right-hand joints, and six face/head-related keypoints. The coordinate or keypoint-value convention was inherited from the original AI Hub annotation format and then transformed into a normalized model-input representation during preprocessing. Therefore, the proposed model should be understood as generating normalized keypoint sequences derived from RGB-video annotations, not as reconstructing metric 3D world coordinates from sensors.

During preprocessing, each pose sequence was normalized to reduce differences in body size among signers and differences in position within the video frame. Specifically, the coordinate center was shifted based on a torso anchor, and the distance between the left and right shoulders was used as the reference scale for coordinate normalization. This allows the model to focus on relative body structure and motion patterns rather than absolute image coordinates. Abnormal or non-finite values were replaced with zeros, and excessively large coordinate values were clipped within a predefined range to ensure training stability. No additional sensor fusion or manual three-dimensional reconstruction was performed in this study.

For temporal processing, the original frame sequence was subsampled using a fixed stride, and the maximum number of frames was limited to 80. In the final experimental setting, we used stride=3 and max_frames=80. Therefore, overly long sign language sequences were truncated to the first 80 sampled frames. Sequences shorter than 80 frames were padded during batch construction. The actual pose length of each sample was stored separately and used to mask padded regions so that they did not affect loss computation or evaluation. Missing or invalid frames were not used to introduce new motion content; invalid coordinate values were replaced during preprocessing, and padded regions were handled only through masking rather than interpolation-based evaluation.

### 3.3. Definition of Non-Manual Signals

Non-manual signals refer to bodily expressions other than hand and arm movements that contribute to meaning delivery in sign language [3,15,22,23]. In this study, non-manual labels were constructed from the non-manual metadata tags included in the original morpheme-level annotations. They were not generated by applying thresholds to the predicted pose coordinates or landmarks. Instead, the annotation tags were grouped into four binary dimensions and converted into a multi-hot vector:

(4) $$n\in {\left\{0,1\right\}}^{K},$$

where *K* denotes the dimension of the non-manual signal vector, and K=4 in this study. The four dimensions correspond to: (1) facial or eyebrow/eye-related expression, (2) mouth or mouthing-related expression, (3) head movement or head orientation, and (4) upper-body or torso-orientation expression. These dimensions were selected because they summarize the major non-manual categories that can be linked to the available metadata and to the selected face/head/upper-body pose joints.

For each sample, a non-manual dimension was assigned a value of 1 when at least one metadata tag belonging to that group was present in the sample-level or segment-level annotation; otherwise, it was assigned a value of 0. Therefore, the ground-truth non-manual vector is a metadata-derived binary multi-label vector. Since a single sign language sample can contain multiple non-manual signals simultaneously, the non-manual signal is represented in a multi-label form rather than as a single mutually exclusive class label.

No coordinate- or landmark-threshold rule was used to create the ground-truth non-manual labels. The binary conversion threshold was therefore defined at the metadata-tag level: the presence of at least one relevant annotation tag was converted to 1, and the absence of such tags was converted to 0.

During evaluation, the model outputs probabilities for the four non-manual dimensions. Fixed-threshold precision, recall, and F1 were computed by binarizing the predicted probabilities using the predefined decision threshold, and best-threshold F1 was additionally reported as a diagnostic measure of threshold sensitivity. In the reviewer-revision focal-weighting experiments, several decision thresholds were examined to analyze the precision–recall trade-off and the tendency of the model to overpredict or underpredict sparse non-manual labels.

The non-manual signals considered in this study can be interpreted from three perspectives. First, face-based signals are related to eyebrow movements, mouth shapes, and facial expression changes, and can be used to convey meanings such as questions, emotions, and emphasis. Second, head-movement-based signals are related to nodding, head shaking, and directional changes, and can support meanings such as sentence type, speaker attitude, negation, or agreement. Third, upper-body-orientation-based signals are related to torso tilting, directional changes, and spatial reference expressions, and can contribute to representing relationships between entities or sentence flow in sign language.

In existing pose generation models, such non-manual signals are often treated only as part of the overall coordinates. In this case, even if the model predicts coordinates in non-manual regions, it is difficult to explicitly learn how these expressions are related to the meaning of the input text. To alleviate this issue, this study uses non-manual signals as separate conditional information. That is, non-manual signals are used not merely as output coordinates, but as auxiliary conditions that connect textual meaning and sign language expression during pose sequence generation.

Through this design, the model predicts the presence of non-manual signals from the input text and reflects the predicted non-manual conditions in the pose generation process. The final generated pose sequence includes not only manual movements centered on the hands and arms, but also facial, head, and upper-body expressions. The goal is to generate pose sequences that better reflect the expressive structure of sign language by jointly modeling these components.

### 3.4. Problem Definition

This study addresses the problem of generating KSL pose sequences from natural language text sentences. The input is a morpheme-level tokenized text sequence, and the output is a temporally aligned sign language pose sequence. A single training sample can be defined as follows:

(5) $$D={\left\{\left(x^{\left(i\right)},Y^{\left(i\right)},n^{\left(i\right)},L^{\left(i\right)}\right)\right\}}_{i=1}^{N},$$

where $x^{\left(i\right)}$ denotes the input text token sequence of the *i*-th sample, $Y^{\left(i\right)}$ denotes the corresponding ground-truth KSL pose sequence, $n^{\left(i\right)}$ denotes the multi-hot vector of non-manual signals, and $L^{\left(i\right)}$ denotes the actual length of the pose sequence.

The input text is represented as follows:

(6) $$x^{\left(i\right)}=\left(x_{1}^{\left(i\right)},x_{2}^{\left(i\right)},\ldots ,x_{M}^{\left(i\right)}\right),$$

where *M* is the input token length and each $x_{m}$ is a token ID in the vocabulary. Conditioned on this input, the model generates the following pose sequence:

(7) $${\hat{Y}}^{\left(i\right)}=\left({\hat{y}}_{1}^{\left(i\right)},{\hat{y}}_{2}^{\left(i\right)},\ldots ,{\hat{y}}_{\hat{T}}^{\left(i\right)}\right),\;{\hat{Y}}^{\left(i\right)}\in R^{\hat{T}\times J\times C},$$

where $\hat{T}$ denotes the output length predicted by the model or used during the generation process. In this study, considering the length mismatch between text and sign language pose sequences, the pose length is predicted together with the pose sequence, and a length blending strategy is applied during training and inference.

The goal of this study is to generate a pose sequence $\hat{Y}$ that spatially corresponds to the ground-truth pose sequence Y while also exhibiting temporally natural and consistent motion. For this purpose, the evaluation is conducted from the following four perspectives.

First, keypoint-level pose accuracy is evaluated. Metrics such as MPJPE, Pose MAE, and Pose RMSE are used to measure how close the generated normalized keypoint sequences are to the ground-truth pose representation. These metrics indicate how accurately the model generates the basic sign language postures and joint locations corresponding to the textual meaning.

Second, pose correctness is evaluated. The PCK metric is used to determine whether generated joints match the ground-truth joints within a predefined error threshold. In this study, multiple threshold-based metrics, such as PCK@0.03, PCK@0.05, and PCK@0.10, are used to analyze both strict and relaxed positional accuracy.

Third, length consistency is evaluated. The length relative error is used to measure how similar the generated pose sequence length is to the actual sign language expression length for the input text. This metric reflects the model’s ability to predict temporal alignment and expression duration between text and sign language.

Fourth, the quality of non-manual signal modeling is evaluated. Metrics such as non-manual F1 are used to assess whether the model appropriately predicts non-manual signals corresponding to the input text. The effect of these predicted signals on pose generation is further analyzed through ablation studies and qualitative comparisons. This evaluation determines the extent to which the generated poses reflect the linguistic characteristics of KSL beyond simple hand-centered motion.

Therefore, the problem addressed in this study is not a simple coordinate regression problem, but a conditional sequence generation problem that simultaneously considers textual meaning, pose accuracy, motion naturalness, length consistency, and non-manual expression. To satisfy these complex requirements, the proposed model generates KSL pose sequences by combining non-manual signal conditioning with a multi-scale temporal refinement structure.

## 4. Proposed Method

### 4.1. Overall Framework

The proposed framework combines non-manual signal conditioning and multi-scale temporal refinement to generate KSL pose sequences from natural language text inputs. This design is motivated by prior sign language production studies that generate pose sequences from text or gloss representations [8,9,10,14], as well as recent text-to-motion and diffusion-based motion generation studies that emphasize temporal modeling and motion refinement [19,20,21,25].

**Figure 2:**
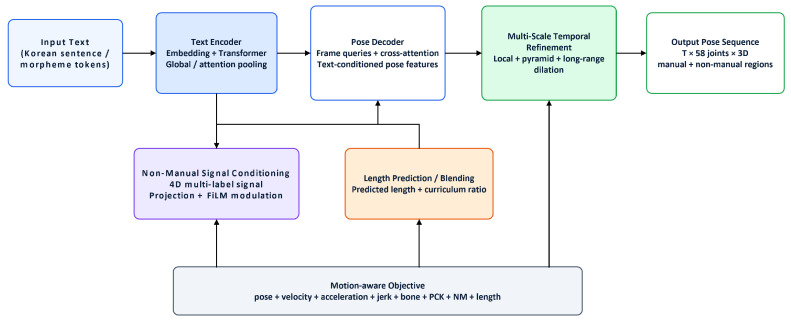
Overall framework of the proposed text-to-KSL pose generation model.

Figure 2 illustrates the overall architecture of the proposed text-to-KSL pose generation model.

The overall framework consists of the following major components: a text encoder, a pose decoder, a non-manual signal conditioning module, a multi-scale temporal refinement module, a length prediction and blending module, and a motion-aware learning objective. The text encoder converts an input token sequence into a sentence-level semantic representation. The pose decoder generates a temporal pose sequence using the text representation and frame queries. The non-manual signal conditioning module predicts non-manual signals from the input text and injects them as conditional information into the pose generation process. The multi-scale temporal refinement module refines the generated pose features over various temporal ranges to capture both local motion patterns and long-term temporal context. The length prediction and blending module predicts the pose sequence length corresponding to the input text and mitigates length mismatch during training and inference. Finally, the motion-aware learning objective jointly optimizes coordinate accuracy, velocity, acceleration, jerk, bone consistency, PCK-based precision, non-manual signals, and length consistency.

Given an input text sequence $x=\{x_{1},x_{2},\ldots ,x_{M}\}$, the proposed model generates the following outputs:

(8) $$\left(\hat{Y},\hat{n},\hat{L}\right)=f_{\theta }\left(x\right),$$

where $\hat{Y}\in R^{\hat{T}\times J\times C}$ denotes the generated KSL pose sequence, $\hat{T}$ denotes the predicted or adjusted output sequence length, *J* denotes the number of joints, and *C* denotes the number of normalized keypoint channels per joint. In this study, we use J=58 and C=3, and thus each frame is represented as a 174-dimensional pose vector.

The key ideas of the proposed model are as follows. First, the model directly generates sign language motions from textual semantic representations while separately predicting non-manual signals and using them as pose generation conditions. Second, the generated pose features are not processed at a single temporal resolution. Instead, they are refined over multiple temporal ranges through local temporal refinement, multi-scale convolution, temporal attention, temporal pyramid fusion, and long-range dilated refinement. Third, length prediction and length blending are applied to alleviate the problem that text length and pose sequence length do not directly correspond to each other. Through these components, the proposed model generates KSL pose sequences that jointly consider manual movements, non-manual expressions, temporal naturalness, and length consistency.

### 4.2. Text Encoder

The text encoder converts an input sentence into a high-dimensional semantic representation that can be used for pose generation. This design follows the general paradigm of neural text representation learning based on token embeddings and contextual encoding [38,49]. During preprocessing, the input sentence is decomposed into morpheme-level or token-level units, and each token is converted into an integer ID according to the vocabulary. The input token sequence is defined as follows:

(9) $$x=(x_{1},x_{2},\ldots ,x_{M}),$$

where *M* denotes the input text length. Each token is converted into a continuous vector through an embedding layer, and a text positional embedding is added to incorporate positional information:

(10) $$e_{m}=\mathrm{Embed}\left(x_{m}\right)+{\mathrm{PE}}_{m}^{\mathrm{text}}.$$

The embedding vectors are then transformed into the hidden dimension of the model through a linear projection. The final model in this study uses a text embedding dimension of 512 and a hidden dimension of 1024. The transformed text feature sequence is passed through a Transformer encoder, where self-attention learns semantic relationships among tokens in the sentence [38]:

(11) $$H=\mathrm{TransformerEncoder}(E,M_{\mathrm{pad}}),$$

where $H\in R^{M\times d}$ denotes the token-level text representation and *d* denotes the hidden dimension. A text-length-based mask is used so that padding tokens do not affect the attention computation.

To obtain a sentence-level semantic representation, two types of summary information are combined from the encoder output. First, average pooling is performed over valid tokens to compute a global representation of the entire sentence. Second, a token attention-based summary is computed to assign larger weights to tokens that are more relevant to non-manual signals:

(12) $$h_{\mathrm{avg}}=\frac{1}{M^{\prime }}\sum_{m=1}^{M^{\prime }}H_{m},$$

(13) $$\alpha_{m}=\mathrm{softmax}\left(w^{⊤}H_{m}\right),\;h_{\mathrm{attn}}=\sum_{m=1}^{M^{\prime }}\alpha_{m}H_{m},$$

where $M^{\prime }$ denotes the number of valid tokens excluding padding. The two summary vectors are combined through a gate:

(14) $$g=\sigma \left(W_{g}\left[h_{\mathrm{avg}};h_{\mathrm{attn}}\right]\right),$$

(15) $$h=g⊙h_{\mathrm{avg}}+\left(1-g\right)⊙h_{\mathrm{attn}}.$$

The resulting vector h is used as a common conditioning vector for the pose generation decoder, the non-manual signal predictor, and the length predictor. Thus, the text encoder not only embeds the input sentence, but also provides a central representation that controls pose length, non-manual signals, and frame-wise pose generation.

### 4.3. Non-Manual Signal Conditioning Module

The non-manual signal conditioning module is designed to explicitly incorporate facial, head, and upper-body expressions, which are important for meaning delivery in KSL, into the pose generation process [3,15,22,23]. Existing pose generation methods often predict facial or upper-body joints only as part of the entire coordinate set. However, such approaches make it difficult to explicitly learn how non-manual signals are related to textual meaning. To address this limitation, this study jointly performs text-based non-manual signal prediction and pose generation conditioning.

**Figure 3:**
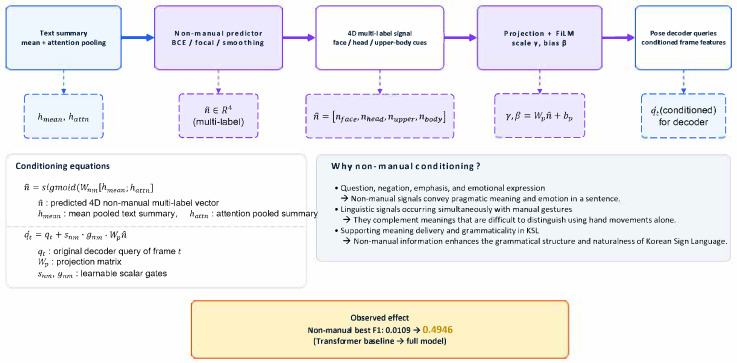
Non-manual signal conditioning module.

Figure 3 illustrates the structure of the non-manual signal conditioning module.

First, the global summary h obtained from the text encoder and the token attention summary $h_{\mathrm{attn}}$ are combined for non-manual signal prediction:

(16) $$z_{\mathrm{nm}}=\phi_{\mathrm{nm}}\left([h;h_{\mathrm{attn}}]\right).$$

The non-manual signal predictor takes $z_{\mathrm{nm}}$ as input and generates multi-label logits:

(17) $$\hat{n}=\sigma \left(W_{\mathrm{nm}}z_{\mathrm{nm}}+b_{\mathrm{nm}}\right),$$

where $\hat{n}\in {\left[0,1\right]}^{K}$ denotes the predicted non-manual signal probabilities. In this study, we use a multi-hot non-manual signal vector with K=4. Since non-manual signals are not mutually exclusive single classes but multiple labels that can occur simultaneously, they are treated as a sigmoid-based multi-label prediction problem.

The predicted non-manual signals are injected into the pose generation process in two ways. First, the non-manual signal vector is projected into the hidden dimension and added to each frame query:

(18) $${\tilde{q}}_{t}=q_{t}+s_{\mathrm{nm}}g_{\mathrm{nm}}W_{p}\hat{n},$$

where $q_{t}$ denotes the *t*-th frame query, $W_{p}$ denotes the non-manual signal projection layer, $g_{\mathrm{nm}}$ denotes a gate that controls conditioning strength, and $s_{\mathrm{nm}}$ denotes the non-manual conditioning scale.

Second, feature modulation is applied using a FiLM-like mechanism, which is commonly used to condition intermediate features on auxiliary information. Scale and bias parameters are generated from the non-manual signals and used to modulate the frame query features:

(19) $$\gamma_{\mathrm{nm}},\beta_{\mathrm{nm}}={\mathrm{MLP}}_{\mathrm{FiLM}}\left(\hat{n}\right),$$

(20) $${\tilde{q}}_{t}=\gamma_{\mathrm{nm}}⊙q_{t}+\beta_{\mathrm{nm}}.$$

Through this mechanism, non-manual signals function not merely as auxiliary outputs, but as conditional information that directly modulates the input features of the pose decoder. In addition, during the early stage of training, teacher forcing is applied by partially using ground-truth non-manual signals. As training progresses, the model gradually uses predicted non-manual signals to reduce the train-test mismatch. In the final model, the strength of non-manual conditioning is increased using a warm-up strategy to prevent unstable pose generation during the early training stage.

### 4.4. Pose Sequence Generation Decoder

The pose sequence generation decoder generates temporal pose features using the text encoder memory and frame queries to which non-manual conditioning has been applied. Instead of generating the output pose sequence one frame at a time in an autoregressive manner, this study constructs frame queries corresponding to the maximum pose length and generates pose features in parallel through a Transformer decoder [38]. Similar Transformer-based structures have been widely used in sign language production and text-to-motion generation [8,10,25,35].

Each frame query is initialized as the sum of a learnable frame embedding and a frame positional embedding:

(21) $$q_{t}^{0}=e_{t}^{\mathrm{frame}}+{\mathrm{PE}}_{t}^{\mathrm{frame}}.$$

The sentence-level semantic vector h, frame progress feature, length information, and text alignment feature are then added to the query. The frame progress feature reflects both the relative position of the current frame and the predicted total length:

(22) $$r_{t}=\left[\frac{t}{\hat{L}},\sin\left(\frac{\pi t}{\hat{L}}\right),\cos\left(\frac{\pi t}{\hat{L}}\right)\right],$$

(23) $$q_{t}=q_{t}^{0}+W_{h}h+W_{r}r_{t}.$$

In addition, query-to-text alignment is performed to reflect which part of the input text is related to each frame query:

(24) $$a_{t,m}={\mathrm{softmax}}_{m}\left(\frac{{\left(W_{q}q_{t}\right)}^{⊤}\left(W_{k}H_{m}\right)}{\sqrt{d}}\right),$$

(25) $$c_{t}=\sum_{m=1}^{M^{\prime }}a_{t,m}H_{m}.$$

The frame queries with non-manual conditioning are then fed into the Transformer decoder:

(26) $$D=\mathrm{TransformerDecoder}(\tilde{Q},H),$$

where $D\in R^{T\times d}$ denotes the frame-level decoded features. The decoder allows each frame to refer to the semantic information of the input text through cross-attention and models inter-frame relationships through self-attention.

The final pose coordinates are generated by passing the decoded features through a pose head:

(27) $${\hat{p}}_{t}=\mathrm{PoseHead}\left(D_{t}\right),$$

where ${\hat{p}}_{t}\in R^{174}$ denotes the flattened pose vector of the *t*-th frame. Since the generated pose includes body, left-hand, right-hand, and face regions, manual and non-manual poses are generated simultaneously.

This study also uses a coarse pose head and a text-pose prior to encourage stable initial pose generation. The coarse pose is an auxiliary path directly predicted from frame queries, whereas the text-pose prior is generated from the query-to-text alignment results. The final pose prediction is obtained by combining the outputs of the main pose head, coarse pose, and text-pose prior in a residual manner:

(28) $${\hat{p}}_{t}={\hat{p}}_{t}^{\mathrm{main}}+\alpha_{\mathrm{coarse}}{\hat{p}}_{t}^{\mathrm{coarse}}+\alpha_{\mathrm{prior}}{\hat{p}}_{t}^{\mathrm{prior}}.$$

This structure strengthens the connection between text conditions and frame-level pose features and reduces instability that may occur when generating long sequences.

### 4.5. Multi-Scale Temporal Refinement Module

A sign language pose sequence includes both local hand movements and long-term motion flow across the entire sentence [10,15,23]. Similar temporal modeling challenges have been studied in human motion generation and video-based pose modeling [19,21,39,40]. Therefore, a pose generation model should consider not only smooth changes between adjacent frames but also motion transitions and expression continuity over long temporal ranges. To this end, this study introduces a multi-scale temporal refinement module.

**Figure 4:**
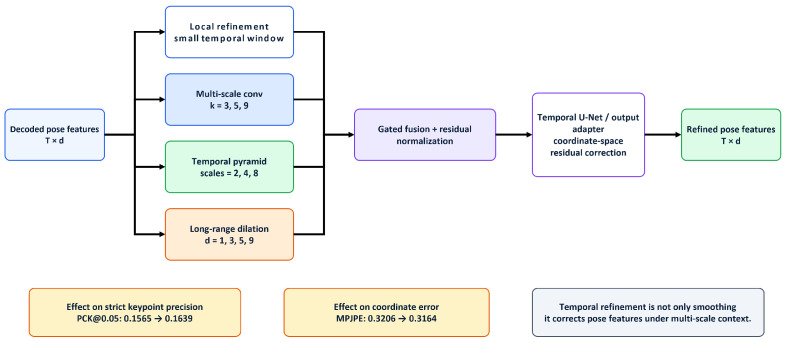
Multi-scale temporal refinement module.

Figure 4 illustrates the structure of the multi-scale temporal refinement module.

The multi-scale temporal refinement module takes the frame-level feature D generated by the Transformer decoder as input and refines it at multiple temporal scales, following the general motivation of local-to-global temporal modeling in motion generation [19,21,39,40]. The proposed temporal refinement module includes local temporal refinement, multi-scale temporal convolution, temporal self-attention refinement, temporal pyramid fusion, gated dilated motion refinement, a long-range temporal context adapter, temporal U-Net-style refinement, and an output-space temporal adapter.

First, local temporal refinement corrects inter-frame continuity over short temporal intervals. It uses depthwise temporal convolution to locally refine temporal changes in each feature channel:

(29) $$D_{\mathrm{local}}=D+{\mathrm{DWConv}}_{k=3}\left(D\right).$$

Next, multi-scale temporal convolution uses multiple temporal branches with different kernel sizes. In the final setting of this study, kernel sizes of 3, 5, and 9 are used to capture both short-term motion changes and relatively longer motion patterns:

(30) $$U_{k}=\mathrm{Conv}1D_{k}\left(D_{\mathrm{local}}\right),\;k\in \left\{3,5,9\right\}.$$

The outputs of each branch are combined using weights computed from the summary feature of the input sequence:

(31) $$\omega_{k}={\mathrm{softmax}}_{k}\left(w_{k}^{⊤}\mathrm{Pool}\left(D_{\mathrm{local}}\right)\right),$$

(32) $$D_{\mathrm{ms}}=\sum_{k\in \{3,5,9\}}\omega_{k}U_{k}.$$

The refined features are then stabilized through residual connection and normalization:

(33) $$D_{\mathrm{ref}}=\mathrm{LayerNorm}\left(D_{\mathrm{local}}+D_{\mathrm{ms}}\right).$$

Temporal self-attention refinement is used to supplement long-term dependencies between frames. It reapplies self-attention to the decoded features after the Transformer decoder so that each frame can refer to contextual information from other frames in the sequence:

(34) $$D_{\mathrm{attn}}=\mathrm{SelfAttention}\left(D_{\mathrm{ref}}\right).$$

Temporal pyramid fusion generates features at different temporal resolutions using pooling scales of 2, 4, and 8, upsamples them back to the original length, and combines them. This structure is used to reflect both short-term and mid- to long-term motion patterns:

(35) $$P_{s}=\mathrm{Upsample}\left(\mathrm{Conv}1D\left({\mathrm{Pool}}_{s}\left(D_{\mathrm{attn}}\right)\right)\right),\;s\in \left\{2,4,8\right\},$$

(36) $$D_{\mathrm{pyr}}=\mathrm{Fuse}\left(D_{\mathrm{attn}},P_{2},P_{4},P_{8}\right).$$

In addition, the long-range temporal context adapter uses dilated depthwise convolution branches with dilation rates of 1, 3, 5, and 9. This structure reflects long-range motion context that is difficult to capture with a short temporal kernel alone:

(37) $$V_{r}={\mathrm{DWConv}}_{k=3,\;d=r}\left(D_{\mathrm{pyr}}\right),\;r\in \left\{1,3,5,9\right\}.$$

(38) $$D_{\mathrm{long}}=\mathrm{Fuse}\left(V_{1},V_{3},V_{5},V_{9}\right).$$

Finally, the temporal U-Net refiner and output-space temporal adapter perform residual correction in the feature space and coordinate space, respectively. The temporal U-Net refiner reflects motion context at intermediate temporal resolutions through a downsample–bottleneck–upsample structure, while the output-space temporal adapter applies a low-strength temporal residual correction to the final predicted coordinates.

This multi-scale temporal refinement structure does not rely on a single temporal module, but refines generated poses across diverse temporal ranges. As a result, the proposed model improves not only frame-wise coordinate accuracy but also the motion continuity and naturalness of the entire sequence.

### 4.6. Length Prediction and Blending Strategy

One important issue in text-based sign language pose generation is that the input text length and the output pose sequence length do not directly correspond to each other [8,10,14]. Even for sentences of the same length, the required number of pose frames may vary depending on the complexity of sign language expression, repeated motions, emphasis, and the duration of non-manual signals. Therefore, if a model uses only a fixed length or excessively depends on the ground-truth length, the generated sequence length and motion speed may become unstable in real inference scenarios.

To address this issue, this study uses a length prediction module. The pose sequence length is predicted from the sentence summary vector h obtained from the text encoder:

(39) $${\hat{L}}_{\mathrm{raw}}={\mathrm{MLP}}_{\mathrm{len}}\left(h\right).$$

In the final model, a sigmoid-based length predictor is used so that the predicted length is constrained between the minimum and maximum length:

(40) $$\hat{L}=L_{\min}+\left(L_{\max}-L_{\min}\right)\cdot \sigma \left({\hat{L}}_{\mathrm{raw}}\right),$$

where $L_{\min}$ and $L_{\max}$ denote the minimum and maximum pose lengths, respectively. In this study, the maximum pose length is set to 80.

During training, the ground-truth pose length is used in the early stage to ensure stable pose generation. After a certain number of epochs, the predicted length is gradually incorporated. For this purpose, a predicted length ratio and a length blending ratio are used. Length blending combines the ground-truth length *L* and the predicted length $\hat{L}$ as follows:

(41) $$L_{\mathrm{blend}}=\left(1-\lambda \right)L+\lambda \hat{L},$$

where λ denotes the length blending ratio. However, since the predicted length may be inaccurate during the early training stage, a sample-wise gate is applied based on the relative error between the predicted and ground-truth lengths:

(42) $$g_{\mathrm{len}}=\exp\left(-\frac{\left|\hat{L}-L\right|}{L+\epsilon }\right),$$

(43) $$L_{\mathrm{blend}}=\left(1-\lambda g_{\mathrm{len}}\right)L+\lambda g_{\mathrm{len}}\hat{L}.$$

Through this strategy, the influence of the predicted length is reduced for samples whose predicted length differs substantially from the ground-truth length, whereas the predicted length is gradually used for samples with stable predictions. During inference, the ground-truth length is unavailable; therefore, the number of frame queries is determined based on the predicted length. During evaluation, a small degree of length blending is applied to mitigate abrupt changes in generated sequence length.

The proposed length prediction and blending strategy reduces temporal mismatch between text and KSL pose sequences and contributes to stabilizing the duration and speed of the generated sign language motions.

### 4.7. Learning Objective

The proposed model combines multiple loss functions to jointly optimize pose coordinate accuracy, motion naturalness, non-manual signal prediction, and length consistency. The overall learning objective is defined as follows:

(44) $$L_{\mathrm{total}}=\lambda_{\mathrm{pose}}L_{\mathrm{pose}}+\lambda_{\mathrm{vel}}L_{\mathrm{vel}}+\lambda_{\mathrm{acc}}L_{\mathrm{acc}}+\lambda_{\mathrm{jerk}}L_{\mathrm{jerk}}+\lambda_{\mathrm{bone}}L_{\mathrm{bone}}+\lambda_{\mathrm{pck}}L_{\mathrm{pck}}+\lambda_{\mathrm{nm}}L_{\mathrm{nm}}+\lambda_{\mathrm{len}}L_{\mathrm{len}}+\lambda_{\mathrm{add}}L_{\mathrm{add}},$$

where each λ denotes the weight of the corresponding loss term.

#### 4.7.1. Pose Reconstruction Loss

The pose reconstruction loss is the primary loss used to reduce coordinate errors between the generated and ground-truth poses. This study uses the Smooth L1 loss, which is relatively robust to outliers:

(45) $$L_{\mathrm{pose}}=\frac{1}{\left|\Omega \right|}\sum_{(t,j)\in \Omega }\mathrm{SmoothL}1\left({\hat{y}}_{t,j},y_{t,j}\right),$$

where Ω denotes the set of valid frames and joints excluding padding regions.

#### 4.7.2. Velocity Loss

The velocity loss encourages the motion changes between adjacent frames to be similar to those of the ground truth:

(46) $$L_{\mathrm{vel}}=\frac{1}{|\Omega_{v}|}\sum_{t\in \Omega_{v}}{∥\Delta {\hat{Y}}_{t}-\Delta Y_{t}∥}_{1},\;\Delta Y_{t}=Y_{t+1}-Y_{t}.$$

This loss alleviates discontinuous or excessively jittery motion, even when the generated poses are accurate at the frame level.

#### 4.7.3. Acceleration Loss

The acceleration loss matches changes in velocity and is used to learn smooth acceleration and deceleration patterns:

(47) $$L_{\mathrm{acc}}=\frac{1}{|\Omega_{a}|}\sum_{t\in \Omega_{a}}{∥\Delta^{2}{\hat{Y}}_{t}-\Delta^{2}Y_{t}∥}_{1},\;\Delta^{2}Y_{t}=\Delta Y_{t+1}-\Delta Y_{t}.$$

#### 4.7.4. Jerk Loss

The jerk loss controls changes in acceleration and reduces excessive tremors or discontinuous movements:

(48) $$L_{\mathrm{jerk}}=\frac{1}{|\Omega_{j}|}\sum_{t\in \Omega_{j}}{∥\Delta^{3}{\hat{Y}}_{t}-\Delta^{3}Y_{t}∥}_{1},\;\Delta^{3}Y_{t}=\Delta^{2}Y_{t+1}-\Delta^{2}Y_{t}.$$

#### 4.7.5. Bone Consistency Loss

The bone consistency loss encourages the distances between adjacent joints, namely bone lengths, to remain similar to those of the ground truth. This loss reduces physically implausible body structures in the generated poses:

(49) $$L_{\mathrm{bone}}=\frac{1}{\left|\Omega_{t}||B\right|}\sum_{t\in \Omega_{t}}\sum_{(u,v)\in B}\left|{∥{\hat{y}}_{t,u}-{\hat{y}}_{t,v}∥}_{2}-{∥y_{t,u}-y_{t,v}∥}_{2}\right|,$$

where *B* denotes the set of bone connections.

#### 4.7.6. PCK-Aware Loss

The PCK-aware loss is used to reduce the discrepancy between the evaluation metric PCK and the training objective. Since pose-based evaluation has been increasingly discussed in sign language production, aligning training objectives with pose-based metrics is important for meaningful evaluation [11]. PCK measures the proportion of joints whose errors are below a specific threshold, but this threshold-based metric is difficult to optimize directly because it is not differentiable. Therefore, this study uses a sigmoid-based surrogate loss to encourage joint distances to become smaller than the threshold:

(50) $$L_{\mathrm{pck}}\left(\tau \right)=1-\frac{1}{\left|\Omega \right|}\sum_{(t,j)\in \Omega }\sigma \left(\frac{\tau -{∥{\hat{y}}_{t,j}-y_{t,j}∥}_{2}}{T_{p}}\right),$$

where τ denotes the PCK threshold and $T_{p}$ denotes the temperature. This study uses a multi-threshold PCK surrogate with thresholds of 0.03, 0.05, and 0.10 to improve both strict and relaxed positional accuracy.

#### 4.7.7. Non-Manual Signal Loss

Non-manual signal prediction is formulated as a multi-label binary classification problem and is trained using binary cross-entropy loss. To alleviate class imbalance in non-manual signals, positive weighting, focal weighting, and label smoothing are jointly applied [50]:

(51) $$L_{\mathrm{nm}}={\mathrm{BCE}}_{\mathrm{weighted}}\left(\hat{n},n\right).$$

In addition, a small-weight entropy regularization term is added to prevent the predicted probabilities from becoming excessively saturated toward one side.

#### 4.7.8. Length Prediction Loss

The length prediction loss encourages the predicted pose length $\hat{L}$ to be close to the actual pose length *L*. This study uses both a Smooth L1-based length loss and a relative length error:

(52) $$L_{\mathrm{len}}=\mathrm{SmoothL}1\left(\hat{L},L\right)+\left|\frac{\hat{L}-L}{L+\epsilon }\right|.$$

This formulation considers not only absolute length errors but also relative length mismatches in both short and long sequences.

#### 4.7.9. Additional Motion and Refinement Losses

The final model additionally includes endpoint pose loss, endpoint velocity loss, velocity cosine loss, frequency loss, diffusion noise prediction loss, diffusion $x_{0}$ reconstruction loss, centered pose loss, velocity delta supervision, and motion focus loss. The diffusion-related losses and refinement strategy are motivated by denoising diffusion, score-based generative modeling, and guidance-based diffusion frameworks [41,42,51]. These losses respectively encourage stable start and end poses, stable start and end velocities, velocity direction consistency, motion pattern consistency in the frequency domain, diffusion refiner learning, centered pose alignment, frame-to-frame delta prediction, and focused learning on regions with large motion.

In summary, the learning objective of this study is not a simple frame-wise keypoint regression loss. Instead, it is designed to jointly optimize pose accuracy, motion continuity, body-structure consistency, PCK-based precision, non-manual expression, and length consistency. Through this objective, the proposed model can generate more accurate and temporally stable KSL pose sequences from text inputs.

## 5. Experimental Setup

### 5.1. Experimental Data Configuration

This study used the AI Hub KSL video dataset as the source data for constructing text–pose pairs [45]. The original AI Hub dataset contains sentence-level and word-level KSL video clips, morpheme-level annotations, non-manual annotation attributes, video metadata, signer information, and frame-level keypoint JSON files. According to the AI Hub data description, the dataset provides sign-language video clips together with morpheme/non-manual annotation files and keypoint values extracted from 30-fps frame images.

In this study, we did not use wearable sensors, data gloves, or depth sensors. Instead, the pose representation was constructed from the keypoint annotations distributed with the AI Hub RGB video dataset. The original keypoint annotations include body, left-hand, right-hand, and face keypoints. These keypoints were converted into the unified 58-joint representation used in this study. Each joint is represented by three annotation-derived keypoint channels. These channels should be interpreted as the normalized model-input keypoint representation derived from the AI Hub annotation files, rather than as metric three-dimensional world coordinates reconstructed by an additional 3D pose-estimation system.

The original AI Hub data include both sentence-level and word-level sign expressions. In the experimental preprocessing, we selected real-captured frontal-view samples and included both sentence-level (SEN) and word-level (WORD) samples. Samples were indexed through three independent manifest files for training, validation, and testing. The manifest files contain the sample identifier, text token IDs, pose file path, non-manual multi-hot vector, pose length, signer metadata, and recording-condition metadata. The split was constructed at the signer level when signer metadata were available, so that the same signer was not intentionally shared across different splits.

The value 16,943 reported for the test set denotes the number of preprocessed test samples after applying the real-captured frontal-view, sentence/word-type, pose-validity, and length-validity filters. It should not be interpreted as the total number of samples in the original AI Hub dataset. Each of these 16,943 samples is one text–pose pair used for inference and metric computation.

The input text is represented as a morpheme-level token ID sequence, and the maximum text length was set to 128. The output pose sequence was limited to a maximum of 80 sampled frames. Samples shorter than the maximum length were padded for batch processing, and a pose mask was applied so that padded regions were excluded from loss computation and evaluation. Invalid or non-finite coordinate values were replaced during preprocessing, and padded regions were handled by masking rather than by interpolation-based evaluation.

The pose data consist of 58 joints, and each joint is represented by three normalized keypoint channels inherited from the AI Hub annotation format. Therefore, the pose dimension of a single frame is defined as follows:

(53) $$D_{\mathrm{pose}}=J\times C=58\times 3=174,$$

where C=3 denotes the number of keypoint channels per joint rather than metric three-dimensional world coordinates reconstructed from sensors.

The entire joint set consists of body, left-hand, right-hand, and face/head-related regions. The body region includes upper-body-centered joints, each hand consists of 21 joints, and the selected face/head-related joints are used to represent non-manual expressions. The pose/keypoint values were derived from the keypoint annotations distributed with the original AI Hub RGB video dataset, not from wearable sensors or data-glove measurements. After converting the provided keypoints into the unified 58-joint format, the keypoint values were normalized by a torso-centered translation and shoulder-distance scaling, and invalid values were handled as described in the pose representation subsection.

The sequence length varies depending on the duration of the original sign language motion, and the model uses the actual pose length of each sample as input information or supervision. During training, the ground-truth length was used to encourage stable pose generation, and the predicted length was gradually incorporated after a certain number of epochs. During inference, the ground-truth length was unavailable; therefore, the pose sequence was generated based on the length predicted by the model’s length predictor.

Table 1 summarizes the experimental data configuration. In the test stage, prediction results were generated for 16,943 samples, and the same preprocessing and evaluation protocol was applied to all comparison models.

### 5.2. Comparison Models

To verify the effectiveness of the proposed model, we configured various comparison models ranging from simple baselines to component-removed ablation models. The purpose of these comparative experiments is to analyze whether the performance improvement of the proposed model is achieved not merely by increasing model size, but by combining key components such as non-manual signal conditioning, multi-scale temporal refinement, motion losses, length prediction, and PCK-aware objectives.

#### 5.2.1. Text-Only Direct Baseline

The text-only direct baseline is the simplest reference model that directly generates a pose sequence from the input text representation. This model does not use complex temporal refinement or non-manual conditioning structures. Instead, it predicts frame-wise pose coordinates based on the encoded text representation. This baseline was used to examine the basic performance of directly regressing pose sequences from text information alone.

#### 5.2.2. Transformer Text-to-Pose Baseline

The Transformer text-to-pose baseline generates pose sequences from input text based on a Transformer encoder–decoder architecture. Compared with the text-only direct baseline, this model more explicitly reflects the sequence-to-sequence structure and models the relationship between text tokens and pose frames using attention. However, this baseline either does not use or only partially uses the core components of the proposed model, such as non-manual signal conditioning, multi-scale temporal refinement, and motion-aware learning strategies. Therefore, it was used as a reference for comparing a general Transformer-based text-to-pose generation structure with the proposed model.

#### 5.2.3. Component-Removed Ablation Models

To analyze the contribution of each component of the proposed model, the following ablation models were constructed:

- **Without non-manual conditioning**: a model without injecting non-manual signals as pose generation conditions.
- **Without non-manual task**: a model without the non-manual signal prediction task.
- **Without temporal refinement**: a model without the multi-scale temporal refinement module.
- **Without motion losses**: a model without motion-related losses such as velocity, acceleration, and jerk losses.
- **Without PCK-aware losses**: a model without PCK surrogate and multi-PCK-based losses.
- **Without length prediction/blending**: a model without length prediction and length blending strategies.
- **Without diffusion refiner**: a model without the diffusion-based refinement module.

These ablation models were evaluated using the same data splits and similar training conditions to analyze the effect of each component on pose accuracy, PCK, length consistency, non-manual signal prediction, and motion naturalness.

#### 5.2.4. Full Proposed Model

The full proposed model is the final model proposed in this study. It includes the text encoder, pose decoder, non-manual signal conditioning module, multi-scale temporal refinement module, length prediction and blending strategy, and motion-aware objective. The final proposed model corresponds to the full configuration including all proposed components.

The overall configuration of the comparison models is summarized in Table 2.

### 5.3. Evaluation Metrics

In this study, we used multiple evaluation metrics to comprehensively assess the quality of generated KSL pose sequences, including coordinate accuracy, threshold-based pose correctness, length consistency, non-manual signal prediction performance, and motion naturalness.

#### 5.3.1. MPJPE

MPJPE, or Mean Per Joint Position Error, measures the average joint-level difference between the generated pose and the ground-truth pose. In this study, it is used as a keypoint-level accuracy metric for the normalized 58-joint pose representation. A lower MPJPE indicates that the generated pose is closer to the ground-truth pose:

(54) $$\mathrm{MPJPE}=\frac{1}{TJ}\sum_{t=1}^{T}\sum_{j=1}^{J}{∥{\hat{y}}_{t,j}-y_{t,j}∥}_{2},$$

where *T* denotes the number of valid frames, *J* denotes the number of joints, and ${\hat{y}}_{t,j}$ and $y_{t,j}$ denote the generated and ground-truth keypoint-channel vectors of the *j*-th joint in the *t*-th frame, respectively.

#### 5.3.2. Pose MAE

Pose MAE represents the mean absolute error between the generated pose and the ground-truth pose. While MPJPE measures the Euclidean distance at the joint level, Pose MAE computes the average absolute error for each normalized keypoint channel:

(55) $$\mathrm{Pose}\;\mathrm{MAE}=\frac{1}{TJC}\sum_{t=1}^{T}\sum_{j=1}^{J}\sum_{c=1}^{C}\left|{\hat{y}}_{t,j,c}-y_{t,j,c}\right|.$$

A lower value indicates more accurate keypoint-channel-level prediction.

#### 5.3.3. PCK@0.05 and PCK@0.10

PCK, or Percentage of Correct Keypoints, measures the proportion of generated joints located within a specific threshold from the ground-truth joint positions. In this study, PCK@0.05 and PCK@0.10 were used as the main metrics, and the stricter PCK@0.03 was also computed:

(56) $$\mathrm{PCK}@\tau =\frac{1}{TJ}\sum_{t=1}^{T}\sum_{j=1}^{J}I\left({∥{\hat{y}}_{t,j}-y_{t,j}∥}_{2}<\tau \right),$$

where τ denotes the threshold. A higher PCK value indicates that more generated joints are located near the ground-truth positions. PCK@0.05 evaluates strict pose precision, whereas PCK@0.10 evaluates pose-structure consistency under a relatively relaxed criterion.

#### 5.3.4. Precision AUC

Precision AUC evaluates the overall positional precision of generated poses by aggregating keypoint precision values computed over multiple thresholds. Since single-threshold PCK can be sensitive to a particular threshold setting, Precision AUC was additionally used to analyze pose quality across diverse error tolerance ranges. A higher value indicates that the generated poses match the ground-truth poses well across multiple thresholds.

#### 5.3.5. Length Relative Error

Length relative error measures the relative difference between the predicted pose sequence length and the ground-truth length:

(57) $$\mathrm{Length}\;\mathrm{Relative}\;\mathrm{Error}=\left|\frac{\hat{L}-L}{L+\epsilon }\right|,$$

where $\hat{L}$ denotes the predicted pose length and *L* denotes the actual pose length. This metric is used to evaluate how effectively the model alleviates the length mismatch between text and sign language pose sequences. A lower value indicates that the model generates a sign language sequence with a more appropriate length for the input sentence.

#### 5.3.6. Non-Manual F1

Non-manual F1 evaluates how accurately the model predicts non-manual signals corresponding to the input text. Since non-manual signals are represented in a multi-label form, precision, recall, and F1-score were computed:

(58) $$F1_{\mathrm{nm}}=\frac{2\cdot {\mathrm{Precision}}_{\mathrm{nm}}\cdot {\mathrm{Recall}}_{\mathrm{nm}}}{{\mathrm{Precision}}_{\mathrm{nm}}+{\mathrm{Recall}}_{\mathrm{nm}}+\epsilon }.$$

In this study, both fixed-threshold non-manual F1 and best-threshold non-manual F1 were reported. The fixed-threshold F1 evaluates the basic prediction performance under a predefined decision threshold, whereas the best-threshold F1 is used as an auxiliary diagnostic metric to analyze the potential performance after threshold adjustment.

#### 5.3.7. Motion Smoothness-Related Metrics

For sign language pose sequences, not only the accuracy of individual frames but also the naturalness of temporal motion is important. Therefore, the following motion-related metrics were also used:

- **Velocity L1**: measures the velocity difference between adjacent frames.
- **Acceleration L1**: measures the difference in acceleration, i.e., changes in velocity.
- **Velocity cosine**: measures the similarity between the generated motion direction and the ground-truth motion direction.
- **Jerk-related loss**: analyzes excessive jitter by controlling changes in acceleration.
- **Frequency loss**: measures differences between generated and ground-truth motion patterns in the frequency domain.

These metrics are used to evaluate whether the generated poses form temporally natural and stable sign language motions, beyond simply being close to the ground-truth coordinates.

### 5.4. Implementation Details

The final model in this study was trained using the full proposed configuration, including non-manual signal conditioning, multi-scale temporal refinement, length prediction and blending, and motion-aware learning objectives. The model was implemented using PyTorch 2.5.1 [52], and training and evaluation were conducted on a single CUDA GPU environment. Mixed precision training was applied, and bfloat16 AMP was used to improve memory efficiency and training efficiency.

The main hyperparameters of the final model are summarized in Table 3.

Training was configured for a maximum of 180 epochs, but early stopping was applied based on the validation selection score. Early stopping was activated after epoch 22, and the patience was set to 45. Training finally stopped at epoch 97, and the best checkpoint was saved based on the validation selection score.

AdamW was used as the optimizer, building on the Adam optimization framework with decoupled weight decay regularization [53,54]. The initial learning rate was set to $3.4\times 10^{-5}$, and the weight decay was set to 0.021. A cosine annealing learning rate scheduler was used, with the minimum learning rate set to $4.4\times 10^{-6}$. To prevent gradient explosion, gradient norm clipping was applied with a threshold of 0.5.

The loss weights were configured to jointly consider pose accuracy, motion naturalness, non-manual signal prediction, and length consistency. The main loss weights are summarized in Table 4.

For multi-scale temporal refinement, four temporal refinement blocks were used, and the temporal kernel sizes were set to 3, 5, and 9. Two temporal attention blocks were used, with eight attention heads. The temporal pyramid scales were set to 2, 4, and 8, and the long-range temporal dilation rates were set to 1, 3, 5, and 9. In addition, the diffusion refiner used a 100-step schedule, and during inference, timestep 25 and strength 0.006 were applied.

Non-manual signal conditioning was not applied abruptly at the beginning of training, but was gradually increased using a warm-up strategy. The non-manual conditioning scale increased up to 0.8, and the warm-up epoch was set to 30. Teacher forcing was used for non-manual signal prediction, with an initial teacher forcing ratio of 0.89 and a minimum ratio of 0.31.

For length prediction, the ground-truth pose length was used during the early training stage, and the predicted length was gradually incorporated after a certain number of epochs. The predicted length during training was used after epoch 13, and length blending was applied after epoch 11. During evaluation, the predicted length was used, and the evaluation length blend ratio was set to 0.085.

Overall, the experimental setup of this study was designed to strengthen the proposed model in three aspects compared with simple coordinate regression models. First, non-manual signal conditioning was used to reflect the linguistic expression elements of KSL. Second, multi-scale temporal refinement was used to jointly model short-term movements and long-term temporal context. Third, motion-aware losses and length prediction were used to optimize not only pose accuracy but also motion naturalness and sequence length consistency.

## 6. Experimental Results and Analysis

### 6.1. Overall Performance Comparison

This section compares the performance of the proposed model with two baseline models and major ablation models. Evaluation was conducted on the test dataset, and keypoint-level pose accuracy, PCK-based pose correctness, motion naturalness, length consistency, and non-manual signal prediction performance were jointly analyzed. The overall quantitative results are presented in Table 5 and Table 6.

**Figure 5:**
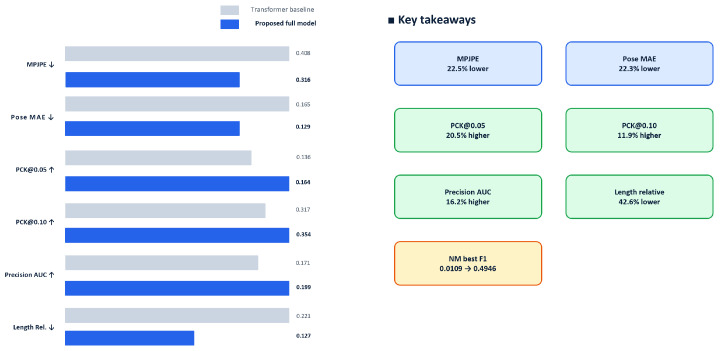
Quantitative comparison with baseline models. Upward arrows indicate higher-is-better metrics, whereas downward arrows indicate lower-is-better metrics.

Figure 5 visualizes the overall quantitative comparison with the baseline models.

The experimental results show that the proposed model consistently outperformed the two baseline models in most major metrics. Compared with the Transformer text-to-pose baseline, the proposed model reduced MPJPE from 0.408236 to 0.316366, corresponding to an error reduction of approximately 22.5%. Pose MAE also decreased from 0.165473 to 0.128570, corresponding to an improvement of approximately 22.3%. These results indicate that the proposed model generates a more accurate normalized keypoint representation than the simple Transformer-based text-to-pose structure.

Improvements were also observed in the PCK-based metrics. PCK@0.05 increased from 0.136090 to 0.163928, and PCK@0.10 increased from 0.316603 to 0.354333. This indicates that the proposed model places more joints near the ground-truth keypoints. In particular, the improvement under the relatively strict threshold of PCK@0.05 shows that the proposed model improves not only the overall pose structure but also detailed joint-position accuracy.

The proposed model also produced more stable results in motion-related metrics than the baselines. Velocity L1 decreased from 7.908108 in the Transformer baseline to 7.273295, and Acceleration L1 decreased from 6.444739 to 6.156408. This indicates that the proposed model generates not only more accurate frame-wise keypoint representations but also more stable inter-frame motion changes.

The improvement in length consistency was particularly notable. The length relative error of the Transformer baseline was 0.221455, whereas that of the proposed model was 0.127152. This corresponds to a relative length error reduction of approximately 42.6%. Therefore, the proposed length prediction and length blending strategies can be interpreted as contributing to more stable prediction of sign language pose sequence lengths corresponding to input text.

A large performance gap was also observed in non-manual signal prediction. The best-threshold non-manual F1 of the Transformer baseline was only 0.010859, whereas the proposed model achieved 0.494566. This suggests that treating non-manual signals as separate learning objectives and conditioning information improves label-level non-manual signal prediction, although it does not by itself verify the full linguistic appropriateness of the predicted non-manual expressions.

Overall, the proposed model achieved better coordinate-based and label-based performance than the baselines in keypoint-level pose accuracy, PCK-based pose correctness, motion stability, length consistency, and non-manual signal prediction. These quantitative results support the usefulness of non-manual signal conditioning and multi-scale temporal refinement for KSL pose sequence generation. However, they should be interpreted as evidence of geometric and temporal improvement rather than as a direct validation that the generated sequences are linguistically complete or recognizable as natural KSL.

### 6.2. Pose Accuracy Analysis

Pose accuracy was analyzed using MPJPE, Pose MAE, Pose RMSE, and PCK metrics. The proposed model achieved an MPJPE of 0.316366, a Pose MAE of 0.128570, and a Pose RMSE of 0.310343, all of which were lower than those of the text-only direct baseline and the Transformer text-to-pose baseline.

Table 7 reports the detailed pose accuracy comparison among the proposed model and baseline models.

In terms of MPJPE, the proposed model reduced the error by approximately 22.5% compared with the Transformer baseline and by approximately 24.5% compared with the text-only direct baseline. In Pose MAE, the proposed model achieved improvements of approximately 22.3% and 24.4%, respectively. These results indicate that the proposed model achieved lower coordinate-level errors than the baseline models under the evaluated test setting.

A similar trend was observed in the PCK metrics. The proposed model achieved a PCK@0.03 of 0.077843, which is higher than 0.060252 obtained by the Transformer baseline. It also achieved PCK@0.05 of 0.163928 and PCK@0.10 of 0.354333, outperforming the baselines at all thresholds. In particular, the improvements in PCK@0.03 and PCK@0.05 indicate that the proposed model generates more accurate poses even under strict joint-position criteria.

Precision AUC was also highest for the proposed model, with a value of 0.198701. Since Precision AUC comprehensively reflects keypoint precision over various error tolerance ranges rather than relying on a single threshold, this result shows that the pose quality of the proposed model was improved overall and was not limited to a specific threshold.

However, in the ablation results, the model without length prediction/blending achieved lower coordinate errors than the full model, with an MPJPE of 0.236135 and a Pose MAE of 0.096704. This suggests that the model may have been in a more favorable condition for coordinate alignment without the burden of length prediction, possibly by relying on ground-truth or fixed-length alignment. However, this setting is less realistic for deployment because it weakens the model’s ability to determine the output sequence length when the ground-truth pose length is unavailable. Although it achieves lower coordinate errors under a more favorable alignment condition, it is less suitable for practical text-to-pose generation than the full model. Accordingly, this study adopts the full model as the final proposed model by considering not only coordinate error but also generated length stability and inference realism.

### 6.3. Analysis of Non-Manual Signal Generation Performance

Non-manual signals play an important role in meaning delivery and expressive naturalness in KSL and sign languages more broadly [3,15,22,23]. In this study, non-manual signal prediction performance was evaluated using fixed-threshold non-manual F1, best-threshold non-manual F1, precision, and recall.

For comparison, all models were evaluated under the same non-manual label space. For models without explicit non-manual conditioning, the available non-manual prediction outputs were used when the corresponding auxiliary prediction branch was included. Therefore, the non-manual F1 comparison should be interpreted as an analysis of explicit non-manual supervision and conditioning, rather than as a direct measure of geometric pose accuracy.

Table 8 summarizes the non-manual signal prediction performance of the comparison models.

The experimental results show that the baseline models achieved extremely low performance in non-manual signal prediction. The best-threshold non-manual F1 of the Transformer baseline was only 0.010859, and that of the text-only direct baseline was also low at 0.009910. This indicates that simple text-to-pose structures have difficulty capturing the relationship between input text and non-manual expressions because non-manual signals are not explicitly learned.

In contrast, the proposed model achieved a fixed-threshold non-manual F1 of 0.470325 and a best-threshold non-manual F1 of 0.494566. Precision and recall were 0.363006 and 0.745283, respectively. The higher recall than precision indicates that the model tends to actively predict non-manual signals. This behavior should be interpreted carefully. Although omitting semantically important non-manual expressions can weaken meaning delivery, predicting unnecessary facial, head, or upper-body signals can also distort meaning, make the generated expression appear unnatural, or cause misunderstanding in real KSL communication. Therefore, the precision–recall balance should be evaluated not only as a metric trade-off but also as a linguistic communication issue.

In the ablation results, the model without the non-manual task achieved a non-manual F1 of zero. This shows that the model cannot explicitly learn non-manual labels when the non-manual signal prediction task is removed. In contrast, the model without non-manual conditioning retained the non-manual signal prediction task but did not use the predicted non-manual signals as pose generation conditions. This model achieved a best-threshold non-manual F1 of 0.488102, slightly lower than 0.494566 of the full model. This suggests that task-level supervision has the largest effect on non-manual signal prediction itself, while conditioning the pose generation process on predicted non-manual signals may further improve pose-level expression patterns in the evaluated setting.

Qualitatively, the model with non-manual signal conditioning tended to show clearer changes in the face, head, and upper-body regions than the baseline models, resulting in improved visual completeness compared with hand-centered pose sequences. In particular, when head movements or upper-body orientation changes were maintained together with manual movements in sentence-level expressions, the generated sequences appeared visually smoother in the selected examples.

### 6.4. Focal Weighting Sensitivity for Non-Manual Signals

Because non-manual labels are sparse and multi-label in nature, focal weighting can substantially affect the balance between precision and recall. To examine whether non-manual overprediction could be alleviated, additional reviewer-revision experiments were conducted using four focal-weighting configurations: a baseline focal setting, a higher precision setting, a balanced F1 setting, and a high recall setting. These settings varied the non-manual BCE weight, focal gamma, class-wise focal alpha values, class-wise positive weights, negative weighting, and fixed decision threshold. The purpose of this sweep was not to identify a universally optimal operating point, but to clarify how calibration choices change false positive and false negative behavior in non-manual signal prediction.

Table 9 summarizes the focal-weighting sensitivity analysis for non-manual signal prediction.

The focal-weighting sweep shows a clear precision–recall trade-off. The baseline focal setting produced a precision of 0.278360 and a recall of 0.364151, indicating that the initial focal configuration did not sufficiently balance sparse non-manual labels. The higher precision setting increased precision to 0.598131 and reduced the predicted positive rate to 0.003158, which is close to the ground-truth positive rate of approximately 0.0037. This result suggests that stronger negative weighting and a higher decision threshold can suppress unnecessary non-manual activations. However, this setting also reduced recall compared with the balanced F1 and high recall settings, meaning that some semantically relevant non-manual signals may be omitted.

The balanced F1 setting achieved the highest fixed-threshold F1 score of 0.633166 with a recall of 1.000000, whereas the high recall setting also achieved a recall of 1.000000 but with a lower F1 score of 0.610169. These settings are useful for showing the upper side of recall-oriented behavior, but they should not be interpreted as naturally optimal settings for actual KSL communication. In a generated sign sequence, excessive non-manual activations may introduce unintended emphasis, affect interrogative or affective nuance, or make the expression less natural. Therefore, the balanced F1 and high recall settings should be understood as metric-oriented operating points that reveal the calibration sensitivity of the model, not as final deployment choices.

The predicted positive rate further supports this interpretation. The baseline focal setting produced a predicted positive rate of 0.008078, which was higher than the ground-truth positive rate of approximately 0.0037. In contrast, the higher precision setting reduced the predicted positive rate to 0.003158, indicating that overprediction can be alleviated by using stronger negative weighting and a higher decision threshold. However, this improvement in precision was accompanied by lower recall than the balanced F1 and high recall settings. Therefore, the focal-weighting results should be interpreted as a calibration analysis rather than as a single best-setting comparison. For practical KSL pose generation, the operating point should be selected according to the communicative cost of two error types: false positive non-manual signals that may distort meaning or naturalness, and false negative non-manual signals that may omit important linguistic information.

### 6.5. Analysis of Temporal Refinement Effects

KSL pose sequences consist of temporally continuous movements; therefore, not only single-frame coordinate accuracy but also inter-frame changes and long-term motion flow are important. To analyze the effect of temporal refinement, this study compared the full model with the model without temporal refinement.

Table 10 reports the effect of the temporal refinement module on pose and motion-related metrics.

The full model achieved better MPJPE and Pose MAE than the model without temporal refinement. MPJPE decreased from 0.320559 to 0.316366, and Pose MAE decreased from 0.130663 to 0.128570. PCK@0.05 also improved from 0.156522 to 0.163928. These results show that the multi-scale temporal refinement module contributes to improving pose accuracy under a strict positional criterion.

However, in PCK@0.10, Velocity L1, and Acceleration L1, the model without temporal refinement showed slightly better values in some cases. This can be interpreted as indicating that temporal refinement is not a module that simply performs smoothing. Instead, it combines features from various temporal scales to correct detailed pose positions. In other words, temporal refinement contributed more directly to improving pose coordinate accuracy and fine-grained PCK performance than to uniformly reducing all motion smoothness metrics.

The multi-scale temporal refinement structure used in this study includes local temporal refinement, multi-scale temporal convolution, temporal attention, temporal pyramid fusion, and long-range dilated refinement. These components were designed to jointly reflect short-term hand movements and sentence-level long-term temporal context. The experimental results show that temporal refinement has a positive effect on pose quality metrics such as MPJPE, Pose MAE, PCK@0.05, and Precision AUC.

From the perspective of motion naturalness, motion losses and temporal refinement can be regarded as complementary components. Temporal refinement corrects poses in the feature and output spaces, whereas motion-aware losses directly constrain temporal derivatives such as velocity, acceleration, and jerk. Therefore, using temporal refinement structures together with motion-aware objectives is appropriate for encouraging more stable temporal behavior in generated pose sequences.

### 6.6. Analysis of Length Prediction and Blending Effects

The length mismatch between text and KSL pose sequences is an important issue in text-to-sign pose generation. Since the input text length and the actual sign language motion length do not directly correspond to each other, the model must predict an appropriate output length according to sentence meaning and expression complexity. To analyze the effect of length prediction and length blending, this study compared the full model with the model without length prediction/blending.

Table 11 shows an important trade-off in the length prediction ablation. The model without length prediction/blending achieved better keypoint-level accuracy and PCK values than the full model. However, its length relative error was 0.208028, which was substantially higher than 0.127152 of the full model. This indicates that removing length prediction/blending can create a favorable frame-alignment condition for coordinate-based evaluation, while weakening the model’s ability to determine the output duration required in realistic text-to-pose inference.

This result should not be interpreted as evidence that length modeling is unnecessary. MPJPE and Pose MAE are computed after temporally comparing generated and ground-truth frames, and are therefore sensitive to frame-alignment conditions. If an ablation model bypasses or weakens length prediction, it may obtain lower coordinate errors because the comparison is performed under a more favorable temporal alignment. This creates a coordinate-metric bias: the model can appear geometrically better even though it is less capable of predicting how long the generated KSL sequence should be when the ground-truth pose length is unavailable.

This diagnostic comparison was computed only on the 14,943 overlapping samples that had valid per-sample records in both the full predicted-length condition and the oracle/no-predicted-length condition. Therefore, the bucket counts in this table differ from the appendix-level pose-length analysis based on the full test set of 16,943 samples.

Because this table was computed using the length-stratified diagnostic evaluation protocol, the absolute MPJPE values should be interpreted for within-table comparison between the full and oracle/no-predicted-length conditions, rather than as directly interchangeable with the overall MPJPE values reported in the main performance tables.

Table 12 provides an additional length-stratified diagnostic analysis using overlapping samples between the full predicted-length condition and the oracle/no-predicted-length condition. The MPJPE gap was small in all buckets, but the sign changed across sequence-length groups. The oracle/no-predicted-length condition was slightly more favorable for medium and long sequences, whereas the full model was slightly better for short sequences. This pattern supports the interpretation that coordinate-based metrics can be influenced by temporal alignment and sequence duration. Therefore, length ablation results should be discussed together with length consistency, rather than being judged only by coordinate error.

The full model substantially reduced the length relative error and produced a more stable temporal correspondence between text and pose sequences. Compared with the Transformer baseline, the length relative error decreased from 0.221455 to 0.127152, corresponding to an improvement of approximately 42.6%. Compared with the text-only direct baseline, it decreased from 0.224558 to 0.127152, corresponding to an improvement of approximately 43.4%.

These results show that the proposed length prediction and blending strategies are not merely auxiliary modules, but play an important role in practical text-to-pose inference. Because the ground-truth pose length is unavailable during deployment, a model must predict an appropriate pose duration from the input text. Therefore, although the ablation model without length prediction/blending appears better when only coordinate fidelity is considered, the full model is more appropriate for realistic text-to-pose generation because it jointly considers coordinate quality, length consistency, and inference feasibility.

From the perspective of sentence length, fixed-length or average-length-based generation may work relatively stably for short sentences. However, as the sentence becomes longer or the sign language expression becomes more complex, the required number of pose frames can vary more substantially, increasing the importance of length prediction. Additional subgroup analysis according to ground-truth pose sequence length is provided in Appendix B.4 to further examine the relationship between sequence duration, coordinate accuracy, and length consistency.

**Figure 6:**
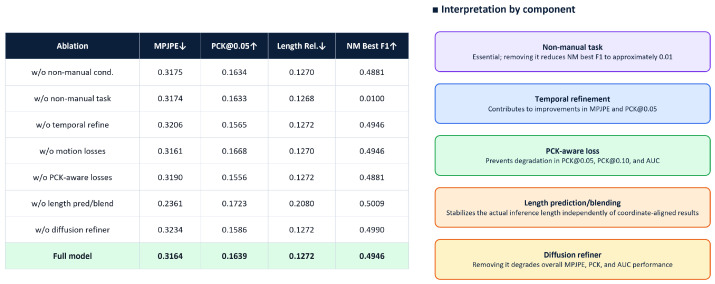
Effects of length prediction and non-manual signal modeling. Upward arrows indicate higher-is-better metrics, whereas downward arrows indicate lower-is-better metrics.

Figure 6 visualizes the effects of length prediction and non-manual signal modeling.

### 6.7. Ablation Study

In this study, an ablation study was conducted to verify the effects of the major components of the proposed model. Table 13 and Table 14 compare the performance of each ablation model and the full model on the test dataset.

**Figure 7:**
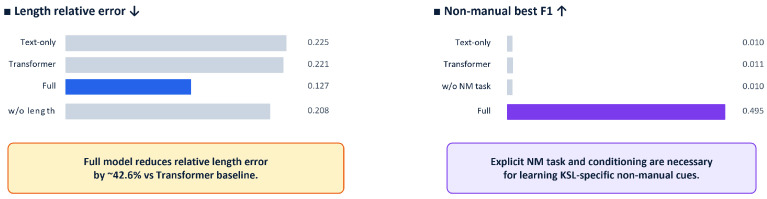
Ablation study results. The bar chart was enlarged with larger bar labels, axis labels, tick labels, and legends to improve readability in the printed PDF.

The ablation results show that each component affects different evaluation metrics. Figure 7 visualizes these ablation study results.

First, regarding the effect of non-manual signal conditioning, the model without non-manual conditioning showed slightly lower performance than the full model in MPJPE, Pose MAE, PCK@0.10, and Precision AUC. Although the difference was not large, the full model generally showed more stable pose accuracy. In addition, best-threshold non-manual F1 increased from 0.488102 to 0.494566. This indicates that using non-manual signals not only as prediction targets but also as pose generation conditions can positively affect overall generation quality.

Second, the model without the non-manual task showed little difference from the full model in pose-related metrics, but its best-threshold non-manual F1 sharply decreased to 0.009960. This indicates that the non-manual signal prediction task is important for learning non-manual expressions. Therefore, explicit supervision of non-manual signals is important in KSL generation models.

Third, removing temporal refinement increased MPJPE to 0.320559 and decreased PCK@0.05 to 0.156522. This shows that the multi-scale temporal refinement module contributes to fine-grained pose position correction. However, some smoothness metrics were better in the model without temporal refinement, suggesting that temporal refinement should be interpreted as a module that corrects pose structure and temporal context rather than simply performing smoothing.

Fourth, the model without motion losses achieved slightly better results than the full model in some coordinate-related metrics, but Velocity L1 and Acceleration L1 degraded to 7.314463 and 6.310742, respectively. This indicates that motion losses are not always advantageous for directly minimizing coordinate errors, but they are necessary for constraining temporal changes and motion naturalness. Therefore, balancing coordinate accuracy and motion naturalness is important in sign language pose generation.

Fifth, removing PCK-aware losses decreased PCK@0.05 from 0.163928 to 0.155599 and PCK@0.10 from 0.354333 to 0.343221. Precision AUC also decreased from 0.198701 to 0.190641. This shows that the PCK-aware objective effectively reduces the gap between the training objective and evaluation metrics and improves threshold-based pose precision.

Sixth, the model without length prediction/blending achieved the best results in pose coordinate metrics but showed a substantially worse length relative error of 0.208028. This indicates a trade-off between keypoint-level pose accuracy and actual generation length consistency. Since the goal of this study is not merely to fit aligned coordinates but to generate sign language sequences with appropriate lengths for input text, length prediction/blending is an important component from the perspective of practical application.

Finally, removing the diffusion refiner increased MPJPE to 0.323396 and decreased PCK@0.05 and PCK@0.10 to 0.158564 and 0.340489, respectively. This indicates that diffusion-based refinement contributes to improving final pose quality and keypoint precision. By including the diffusion refiner, the full model showed more stable performance in coordinate error and PCK metrics.

In summary, the ablation study shows that each component of the proposed model contributes to performance improvement in different aspects. The non-manual task is essential for non-manual signal prediction, the PCK-aware loss improves keypoint precision, and temporal refinement and the diffusion refiner contribute to correcting pose structure and temporal context. Length prediction/blending substantially improves the stability of generated sequence length, independently of coordinate alignment metrics. Therefore, the full model should be interpreted not as a model that always achieves the best performance in every single metric, but as a balanced model that jointly achieves pose accuracy, motion naturalness, length consistency, and non-manual expression quality.

### 6.8. Qualitative Result Analysis

For qualitative analysis, the generated pose sequences for test samples were visualized as skeleton snapshots, joint trajectories, GIFs, and MP4 videos. The visualization results were used to compare the differences between the baseline models and the proposed model not only at the frame level but also from the perspective of temporal motion flow.

In the qualitative results, the baseline models could generate the overall shape of sign language motions, but in some samples, hand positions were unstable or joint locations fluctuated during motion transition intervals. In particular, for long sentence-level sign language expressions, weak connectivity between preceding and subsequent motions or abrupt changes in hand or arm positions were observed in certain frames. This can be attributed to the limited ability of simple text-to-pose structures to sufficiently reflect long-term temporal context and motion continuity.

In contrast, the proposed model tended to maintain the overall flow of pose sequences more stably through the multi-scale temporal refinement structure. Local temporal refinement alleviated abrupt changes between adjacent frames, while multi-scale temporal refinement and long-range temporal modeling contributed to preserving the motion flow across the entire sentence. In the visualized skeleton sequences, the proposed model showed relatively smoother trajectories of hands and arms, and the motion onset and ending intervals appeared more stable.

In terms of non-manual signals, the proposed model showed clearer changes in the face, head, and upper-body regions than the baseline models. In particular, the model with non-manual conditioning tended to generate not only hand movements but also head movements and upper-body orientation changes together with manual motions. This can be interpreted as the effect of injecting non-manual signals as generation conditions rather than treating them merely as part of the coordinate output.

**Figure 8:**
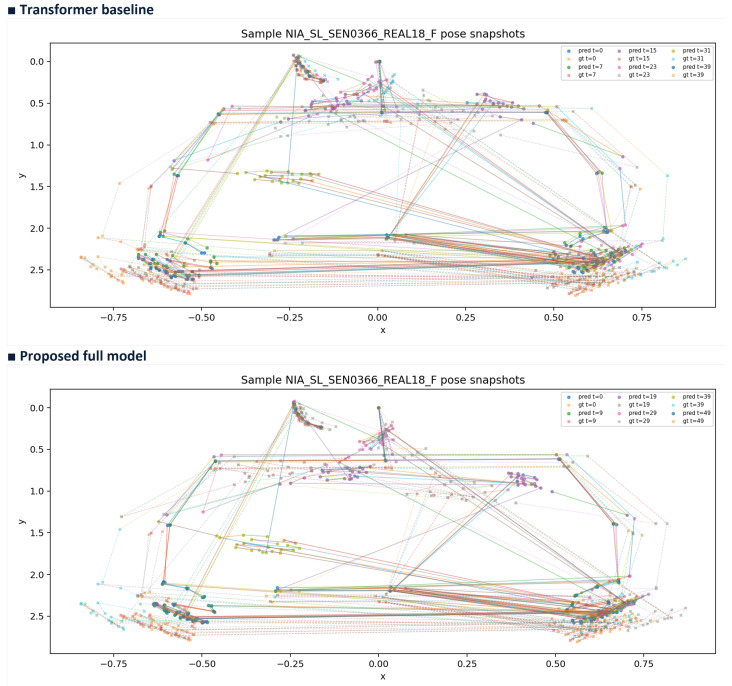
Qualitative pose sequence comparison. The figure scale was increased to improve the readability of pose-sequence snapshots and internal labels in the PDF version.

Figure 8 compares snapshots of generated pose sequences from the baseline and proposed models. Each panel represents the generated result of a different model, and selected temporal snapshots are overlaid in the same coordinate space to illustrate the evolution of pose structure over time. This comparison visually illustrates that the proposed model maintains the overall structure and temporal flow of sign language motions more stably.

**Figure 9:**
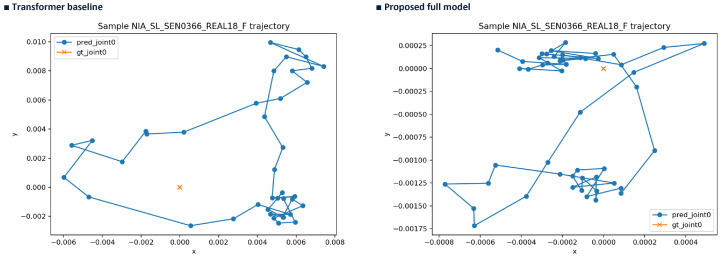
Example of joint trajectory comparison.

Figure 9 visualizes the trajectory of a specific joint and compares the motion paths across models. In the baseline model, the joint trajectory tends to show irregular or sharply turning segments, whereas the proposed model tends to maintain a trajectory more similar to the ground truth. This supports the contribution of velocity- and acceleration-based learning objectives and temporal refinement to stabilizing motion trajectories.

For samples containing non-manual signals, the qualitative results were used to examine how changes in the face, head, and upper-body regions were combined with hand movements. In particular, comparisons with baseline or ablated results help illustrate that the proposed model more explicitly reflects the linguistic expression elements of KSL.

The qualitative analysis is generally consistent with the quantitative results in that the proposed model showed more stable pose structures and temporal flows in the selected visualizations. However, these visualizations should be interpreted as illustrative examples rather than as evidence that the generated sequences are fully recognizable as KSL. In addition, the improvement in non-manual F1 suggests that non-manual signals are more explicitly predicted, but it does not guarantee that their timing, intensity, or linguistic function is always appropriate.

However, several limitations were also observed. For long sentences or complex sign language expressions, detailed finger positions still differed from the ground truth in some cases, and facial and upper-body expressions did not fully reproduce subtle non-manual signals of real signers. In addition, considering that non-manual precision was lower than recall, the model may tend to overpredict non-manual signals. Therefore, future work should more precisely annotate the occurrence timing and duration of non-manual signals and incorporate expert-based semantic accuracy evaluation by sign language specialists.

Overall, the quantitative and visualization-based qualitative results suggest that the proposed model produces lower coordinate errors and more temporally stable pose trajectories than the baseline models in the evaluated test setting. In particular, non-manual signal conditioning and multi-scale temporal refinement were found to be effective in jointly reflecting important expressive elements and temporal motion structures in KSL generation.

### 6.9. Error Analysis

To further analyze the remaining failure cases, we examined prediction errors according to pose-sequence length, sample-level failure rankings, non-manual prediction behavior, and signer variability. This analysis complements the aggregate metrics by identifying when and why the model tends to degrade.

First, longer pose sequences were more difficult than short or medium sequences in terms of coordinate accuracy. Although a reliable short/medium/long comparison based on input text length was not conducted because the morpheme-level token-length distribution was concentrated in the short-text group, long sentence-level expressions were indirectly reflected through longer ground-truth pose sequences. In the reviewer-revision analysis, the short group (≤30 frames) had a mean MPJPE of 0.201294, whereas the medium group (31–55 frames) and long group (>55 frames) had mean MPJPE values of 0.293494 and 0.345619, respectively. This trend indicates that long pose sequences require more difficult motion transitions and long-range temporal consistency. However, length mismatch showed a different pattern: short pose sequences were more vulnerable to relative length errors because even a moderate absolute length difference can become large when divided by a short ground-truth sequence length. Therefore, coordinate error and length error should be interpreted as complementary failure modes rather than as a single unified trend.

Second, the largest coordinate errors were often observed in samples with substantial length mismatch or rapid motion changes. For example, the highest-error samples included cases where the predicted pose length was much longer or shorter than the ground-truth length. Such cases are particularly problematic because temporal misalignment can increase MPJPE even when some local poses are plausible. This supports the need to evaluate length consistency together with coordinate-based metrics.

Third, hand and finger regions remained more error-prone than stable upper-body regions. Detailed finger articulation is difficult to reproduce from sentence-level text alone because small differences in finger configuration can have large linguistic effects but occupy a small portion of the coordinate loss. The failure cases also suggest that rapid hand motion and transitions between adjacent signs can produce abrupt deviations in wrist or fingertip trajectories.

Fourth, non-manual signals showed a precision–recall trade-off. The model tended to actively predict non-manual labels, which is useful for avoiding omission of semantically important expressions. However, false positives can occur when facial, head, or upper-body expressions are weak or absent. The focal-weighting sweep showed that the higher precision setting reduced the predicted positive rate and alleviated overprediction, whereas the balanced F1 and high recall settings increased recall but retained more positive activations. This result suggests that non-manual calibration requires more detailed frame-level annotation and expert review rather than only changing the classification threshold.

Finally, subject variability affected generation difficulty. The reviewer-revision subject analysis included three signer groups and showed a subject-level MPJPE standard deviation of 0.053629. The highest subject-level mean MPJPE was 0.385231, whereas the lowest was 0.269585. These differences suggest that signer-specific motion amplitude, body proportions, signing speed, and facial-expression intensity can influence pose-generation performance even when the same preprocessing and model are used.

These failure modes indicate that the remaining errors are not caused by a single component. Rather, they arise from the interaction among temporal alignment, detailed hand articulation, non-manual calibration, signer-dependent motion style, and the intrinsic limitation of coordinate-based evaluation. Therefore, future improvements should combine signer-aware modeling, more detailed hand and facial annotations, frame-level non-manual supervision, and human expert evaluation.

## 7. Discussion

### 7.1. Effect of Non-Manual Signal Conditioning

A central objective of this study is to explicitly incorporate non-manual signals into KSL pose generation. Sign language does not convey meaning solely through hand and arm movements. Non-manual signals, such as facial expressions, head movements, eye gaze, and upper-body orientation, play important roles in expressing sentence type, emphasis, negation, emotion, and the speaker’s attitude [3,15,22,23]. Therefore, for a text-based sign language pose generation model to generate results close to real sign language expressions, it should consider not only manual movements but also non-manual expressions.

The experimental results of this study quantitatively demonstrate this necessity. The text-only direct baseline and Transformer text-to-pose baseline achieved very low best-threshold non-manual F1 scores of 0.009910 and 0.010859, respectively. This indicates that a general text-to-pose structure can hardly capture the relationship between input text and non-manual expressions when non-manual signals are not explicitly learned. In contrast, the proposed model achieved a best-threshold non-manual F1 score of 0.494566, showing a substantial improvement over the baselines. This result suggests that learning non-manual signals as a separate prediction task and injecting them as conditioning information can improve label-level modeling of non-manual expressions, although it does not by itself verify the full linguistic appropriateness of the generated KSL sequences.

The ablation results also support the importance of non-manual signals. The model without the non-manual task showed a sharp decrease in best-threshold non-manual F1 to 0.009960. This indicates that it is difficult for the model to stably learn non-manual expressions without including non-manual signal prediction as an explicit learning objective. In contrast, the model without non-manual conditioning achieved a best-threshold non-manual F1 of 0.488102, which was slightly lower than that of the full model. This result suggests that the non-manual signal prediction task itself has the most direct effect on learning non-manual expressions, whereas using the predicted non-manual signals as pose generation conditions plays a complementary role in improving overall pose generation quality and expression consistency.

The qualitative results further support the effectiveness of non-manual conditioning. Compared with the baselines, the model with non-manual conditioning tended to show clearer movements in the face, head, and upper-body regions. In particular, in sentence-level expressions, head movements or upper-body orientation changes were observed to be maintained together with hand movements. This suggests that the proposed model can reflect some pose-level patterns associated with non-manual expression, although expert evaluation is still required to determine whether these patterns are linguistically appropriate in KSL.

However, non-manual precision was lower than recall. The proposed model achieved a non-manual recall of 0.745283, whereas its precision was 0.363006. This indicates that the model tends to actively detect non-manual signals and may overpredict them in some samples. This issue is important beyond the metric level. In KSL communication, unnecessary facial, head, or upper-body signals can change emphasis, pragmatic nuance, or sentence type, and may make the generated pose sequence less natural or potentially misleading. Therefore, non-manual prediction should be calibrated with respect to both precision and recall, and future research should model occurrence timing, duration, intensity, and expert-validated semantic appropriateness in greater detail.

### 7.2. Effect of Multi-Scale Temporal Refinement

KSL pose sequences consist of temporally continuous motions. Therefore, a generation model should not only accurately predict joint coordinates in individual frames but also stably maintain inter-frame changes and the overall motion flow of the sentence. In short word-level signs, accurately generating hand positions and motion trajectories is important. In long sentence-level signs, naturally connecting transitions, repetitions, pauses, and ending segments across multiple motions becomes more important.

To reflect these characteristics, the proposed multi-scale temporal refinement module combines local temporal refinement, multi-scale temporal convolution, temporal attention, temporal pyramid fusion, and long-range dilated refinement. It was designed to alleviate abrupt changes between adjacent frames over short temporal ranges and to reflect motion flow and contextual relationships across the entire sentence over long temporal ranges. Through this design, the model can generate more stable sequence representations than baselines that generate poses at a single temporal resolution.

In the experimental results, the effect of temporal refinement was mainly observed in pose accuracy and fine-grained keypoint precision. Compared with the model without temporal refinement, the full model reduced MPJPE from 0.320559 to 0.316366 and Pose MAE from 0.130663 to 0.128570. In addition, PCK@0.05 improved from 0.156522 to 0.163928. These results show that the multi-scale temporal refinement module contributes to generating more accurate keypoints under strict positional criteria by refining generated pose features within temporal context.

However, the model without temporal refinement showed slightly lower values in velocity L1 and acceleration L1. This result suggests that temporal refinement does not operate merely as a smoothing module. In other words, multi-scale temporal refinement corrects keypoint positions by jointly considering pose structure and temporal context, rather than uniformly reducing all temporal derivative errors. In contrast, motion smoothness terms such as velocity, acceleration, and jerk are more directly related to the motion-aware objective. Therefore, it is appropriate to use temporal refinement structures together with motion-aware objectives to improve temporal naturalness.

The role of multi-scale temporal refinement can be interpreted differently for short motions and long sentence-level motions. For short sign expressions, local temporal refinement and small temporal kernels are important for correcting detailed hand and arm movements. In contrast, for long sentences, temporal pyramid fusion and long-range dilation contribute to maintaining motion transitions and the overall sentence-level flow. In this respect, the proposed multi-scale temporal refinement can be regarded as a suitable structure for KSL pose generation.

The qualitative results also showed that the full model produced relatively stable hand and arm trajectories compared with the baselines, with fewer abrupt discontinuities during motion transitions. This indicates that multi-scale temporal refinement contributed to improving the visual naturalness of pose sequences. Since maintaining the overall flow of the sequence is particularly important for long sentence-level samples, structures that reflect long-term temporal context can be important components in future sign language generation research.

### 7.3. Limitations

Although this study improved text-based KSL pose sequence generation by combining non-manual signal conditioning and multi-scale temporal refinement, it has several important limitations in research design, evaluation scope, and interpretation of linguistic quality.

First, there is a limitation in the pose representation. This study used normalized pose/keypoint sequences consisting of 58 joints and three channels per joint as the generation target. Such pose-based representation has the advantages of being easier to train and evaluate than video generation and can be used as an intermediate representation for sign language avatars or subsequent video synthesis models [9,10,11,12,13]. However, pose coordinates alone cannot sufficiently represent high-resolution visual information, such as detailed hand shapes, subtle finger bending, fine changes in facial expressions, and mouth shapes. In sign language, subtle differences in finger configuration and facial expression can directly affect semantic distinctions; therefore, pose representation alone cannot fully replace real sign language expression.

Second, there is a representational gap between real sign language videos and pose sequences. Pose sequences provide structural information about sign language motions, but they do not include visual elements observed in real videos, such as skin appearance, hand shape, facial expression, eye gaze, clothing, depth perception, and motion blur. Therefore, the generated results of this study should be interpreted as a preceding step or intermediate representation for sign language video generation. To generate sign language content that can be provided to actual users, further research is required to combine the proposed pose sequences with avatar rendering or video synthesis models. Recent latent diffusion-based visual generation frameworks also suggest a possible direction for extending pose-based representations toward high-quality visual synthesis [12,13,31,55].

Third, the quality and granularity of non-manual signal labels are limited. In this study, non-manual tags provided in the original annotations were used in a multi-label form. However, non-manual signals should ideally be annotated more precisely in terms of temporal occurrence, duration, intensity, and detailed facial regions, rather than simply indicating their presence at the sample level. The current sample-level or low-dimensional non-manual labels may limit the model’s ability to precisely learn the occurrence timing and synchronization of non-manual signals. This limitation may also be related to the result that non-manual recall was high but precision was relatively low.

Fourth, evaluating sign language grammar and semantic accuracy remains challenging. This study evaluated the model using quantitative metrics such as MPJPE, Pose MAE, PCK, non-manual F1, and length relative error. These metrics are useful for measuring spatial similarity, temporal consistency, and label-level non-manual prediction, but they cannot fully verify whether the generated sequence is linguistically meaningful, grammatically natural, or recognizable as KSL to actual users [5,6,11]. The same meaning can be expressed in sign language in multiple valid ways, and an expression may be semantically acceptable even if it differs from the ground truth in coordinates. Conversely, a sequence with low coordinate error may still fail to convey the intended meaning if hand shape, timing, non-manual expression, or grammatical structure is incorrect. Therefore, the present results should be interpreted as coordinate- and label-level evidence, not as a complete validation of KSL linguistic quality.

Fifth, the evaluation is limited to a single dataset and a single held-out test setting. Although a signer-level split was used when signer metadata were available, this study does not provide a complete subject-independent benchmark over all possible signer identities, recording conditions, or KSL domains. Cross-dataset validation was not conducted, and therefore the generalization ability of the model to other KSL corpora, camera setups, signer populations, or annotation protocols remains unverified. Real KSL expressions vary by signing style, motion amplitude, body shape, arm length, dominant-hand habit, facial-expression intensity, and signing speed. In the additional subject-variability analysis, three signer groups showed different error levels, with subject-level MPJPE ranging from 0.269585 to 0.385231 and a subject-level standard deviation of 0.053629. This suggests that signer-specific style can affect pose generation and should be modeled and evaluated more explicitly.

Sixth, this study did not conduct back-translation evaluation, KSL expert evaluation, or user evaluation. Although a KSL expert evaluation protocol was prepared to assess semantic understandability, motion naturalness, non-manual appropriateness, temporal consistency, and overall acceptability, recruiting KSL experts or certified interpreters within the revision period was not feasible. We also did not use a sign language recognition or translation model to compute a back-translation score. Therefore, the present evaluation remains centered on coordinate-based and label-based metrics. This is a major limitation because MPJPE, PCK, length error, and non-manual F1 cannot fully verify whether the generated signing is semantically understandable, linguistically acceptable, or natural to real KSL users.

Finally, the proposed framework should be positioned as a technical step toward text-to-pose KSL generation rather than as an accessibility system ready for deployment. Actual accessibility requires that generated signing be understandable, culturally and linguistically acceptable, robust across users and contexts, and safe to present in real communication settings. The current experiments do not yet establish those conditions. Future deployment-oriented work should therefore combine pose generation with avatar or video rendering, cross-dataset testing, back-translation or semantic consistency evaluation, and structured assessment by Deaf users, KSL interpreters, and KSL linguists.

### 7.4. Future Work

Based on the results of this study, future research can be extended in the following directions.

First, it is necessary to extend pose sequence generation to sign language video generation. Since the output of this study is a normalized pose/keypoint sequence, an avatar rendering or pose-to-video generation stage is required to use it as visual sign language content that can be provided to actual users. By controlling an avatar using the pose sequences generated by the proposed model or combining them with diffusion-based video generation models, the proposed framework can be extended into an end-to-end sign language generation pipeline from text to sign language video [12,13,31,32,33,55].

Second, signer-aware generation is needed. Real sign language expressions can vary depending on the signer’s body proportions, motion speed, expression habits, and facial expression style. Although this study considered signer-level generalization, it did not explicitly control the style of individual signers. Future work can introduce signer embeddings, style tokens, or motion priors to reflect the expression style of a specific signer for the same text or to generate personalized sign language avatars.

Third, gloss-free text-to-sign generation should be further advanced. Existing sign language generation studies often use glosses as intermediate representations, but gloss annotation is costly and requires language-specific expertise. This study follows the direction of directly generating pose sequences from text, which has recently been explored in gloss-free or text-driven sign language production studies [14,31,32,33]. In the future, large language models, semantic role information, grammatical structure analysis, and multimodal pretraining can be used to develop models that generate more semantically accurate sign language poses without glosses. This direction is related to broader multimodal representation learning, in which language supervision is used to learn transferable representations across modalities [56].

Fourth, user evaluation and expert evaluation are necessary. Coordinate-based metrics are useful for objectively comparing model performance, but they cannot sufficiently evaluate the comprehensibility, naturalness, grammaticality, and semantic fidelity of real sign language expressions. In this revision, a formal expert evaluation has not yet been conducted because recruiting KSL experts within the revision period was not feasible. Future studies should therefore conduct structured evaluations with deaf users, sign language interpreters, and KSL experts. Evaluation criteria should include semantic understandability, motion naturalness, appropriateness of non-manual signals, temporal consistency, sentence grammaticality, and overall acceptability.

Fifth, more detailed non-manual signal annotations are required. This study used limited-dimensional multi-label non-manual signals, whereas real non-manual expressions are temporally dynamic and exhibit diverse patterns across detailed facial and body regions. Future work should annotate facial landmarks, eye gaze, eyebrow movements, mouth shapes, head direction, and upper-body rotation at the segment or frame level so that the occurrence timing and duration of non-manual signals can be learned more precisely. In addition, explicitly modeling the relationships between non-manual signals and sentence type, emotion, and pragmatic meaning can improve the linguistic completeness of sign language expressions.

Sixth, evaluation metrics should be expanded. The currently used MPJPE, Pose MAE, PCK, length relative error, and non-manual F1 are useful for measuring geometric accuracy and some aspects of expression quality. However, in sign language generation, factors such as semantic preservation, grammatical validity, temporal rhythm, hand-shape accuracy, and synchronization of non-manual signals are also important. In future work, a more comprehensive evaluation framework should be established by introducing back-translation scores using sign language recognition models, gloss consistency, semantic similarity, motion realism scores, and expert ratings [5,11,27,29].

In summary, this study suggests the potential of non-manual signal conditioning and multi-scale temporal refinement for text-based KSL pose sequence generation. Future studies should further examine whether the proposed framework can be extended toward user-facing KSL generation systems by moving beyond pose representation to avatar or video generation and by incorporating signer style, detailed non-manual annotations, cross-dataset validation, and expert/user evaluation.

## 8. Conclusions

This study proposed a text-to-KSL pose generation model that combines non-manual signal conditioning, multi-scale temporal refinement, motion-aware learning objectives, and length prediction/blending. The goal was to generate normalized 58-joint KSL keypoint sequences from morpheme-level text inputs while considering both manual movements and non-manual expressions.

The experimental results show meaningful empirical improvements over text-only and Transformer-based baselines in coordinate-based pose accuracy, PCK-based pose correctness, motion-related metrics, length consistency, and non-manual signal prediction. These results support the usefulness of explicitly modeling non-manual signals and temporal context for text-based KSL pose generation. Nevertheless, these metrics evaluate geometric and label-level properties of generated pose sequences and do not fully guarantee linguistic meaningfulness, grammatical acceptability, or recognizability as natural KSL.

The results also reveal important trade-offs. The model without length prediction/blending achieved lower coordinate errors than the full model, but this advantage can be partly explained by a more favorable frame-alignment condition and was accompanied by weaker length modeling. Because practical text-to-pose inference requires predicting output duration without access to the ground-truth pose length, the full model was selected by considering both pose fidelity and length consistency. Similarly, non-manual signal prediction showed a calibration-sensitive precision–recall trade-off: high-recall settings can reduce omissions, but unnecessary non-manual activations may distort meaning, reduce naturalness, or cause misunderstanding in KSL communication.

Several limitations remain. The generated output is a pose sequence rather than a complete sign language video, detailed finger articulation and subtle facial expressions are still difficult to reproduce, and subject variability can affect performance. In addition, this study was evaluated on a single dataset and held-out split, without cross-dataset validation, back-translation scoring, KSL expert evaluation, or user evaluation. Therefore, the proposed framework should be understood as a technical step toward text-to-pose KSL generation, rather than as a deployable accessibility system.

In summary, this study suggests that text-based KSL pose generation should be evaluated not only by frame-wise keypoint accuracy, but also by length consistency, temporal stability, signer variability, non-manual calibration, and ultimately human-centered linguistic evaluation. Future work should extend the pose representation to avatar or video generation, incorporate signer-aware modeling, refine frame-level non-manual annotations, and evaluate semantic understandability, naturalness, temporal consistency, and non-manual appropriateness with KSL experts and users.

## Figures and Tables

**Table 1:** Experimental data configuration.

Item	Setting
Source dataset	AI Hub KSL video/keypoint annotations
Selected data type	Real-captured frontal-view sentence/word samples
Input	Morpheme-level text token sequence
Output	Normalized 58-joint KSL keypoint sequence
Max text length	128
Max pose length	80 sampled frames
Number of joints	58
Keypoint channels per joint	3
Pose dimension per frame	174
Non-manual label dimension	4
Split strategy	Manifest-based train/validation/test split; signer-level separation when available
Test samples after preprocessing	16,943

**Table 2:** Configuration of comparison models.

Category	Model Setting	Purpose
Baseline 1	Text-only direct baseline	Direct text-based pose generation
Baseline 2	Transformer text-to-pose baseline	Transformer-based text-to-pose generation
Ablation	Without non-manual conditioning	Effect of non-manual conditioning
Ablation	Without non-manual task	Effect of non-manual prediction task
Ablation	Without temporal refinement	Effect of temporal refinement module
Ablation	Without motion losses	Effect of motion losses
Ablation	Without PCK-aware losses	Effect of PCK-aware losses
Ablation	Without length prediction/blending	Effect of length prediction/blending
Ablation	Without diffusion refiner	Effect of diffusion refiner
Proposed	Full proposed model	Final proposed configuration

**Table 3:** Implementation and training details.

Item	Setting
Model setting	Full proposed model
Task	Text-to-pose
Vocabulary size	3300
Max text length	128
Max pose length	80
Pose dimension	174
Number of joints	58
Non-manual dimension	4
Hidden dimension	1024
Text embedding dimension	512
Transformer layers	8
Attention heads	16
Feed-forward dimension	4096
Dropout	0.17
Batch size	160
Optimizer	AdamW
AdamW betas	(0.9,0.95)
Learning rate	$3.4\times 10^{-5}$
Minimum learning rate	$4.4\times 10^{-6}$
Weight decay	0.021
Scheduler	CosineAnnealingLR
Epochs	180
Actual stopped epoch	97
Early stopping patience	45
Early stopping metric	Validation selection score
Gradient clipping	0.5
AMP	bfloat16
EMA decay	0.99984
Number of workers	8

**Table 4:** Main loss weights.

Loss Term	Weight
Pose Huber	1.00
Pose precision Huber	0.40
Velocity Huber	0.88
Acceleration Huber	0.25
Jerk loss	0.075
Bone length Huber	0.035
Motion focus Huber	0.045
PCK surrogate	0.14
Multi-PCK surrogate	0.08
Non-manual BCE	1.90
Non-manual entropy	0.0058
Length Huber	0.07
Length relative	0.06
Frequency loss	0.014
Endpoint Huber	0.06
Endpoint velocity	0.04
Diffusion noise	0.045
Diffusion $x_{0}$	0.04
Centered pose Huber	0.15
Velocity delta	0.018

**Table 5:** Overall comparison of pose accuracy and length consistency on the test dataset.

Model	MPJPE ↓	Pose MAE ↓	PCK@0.05 ↑	PCK@0.10 ↑	Precision AUC ↑	Length Rel. ↓
Text-only direct baseline	0.419182	0.170056	0.139028	0.322848	0.173754	0.224558
Transformer text-to-pose baseline	0.408236	0.165473	0.136090	0.316603	0.170982	0.221455
Proposed full model	**0.316366**	**0.128570**	**0.163928**	**0.354333**	**0.198701**	**0.127152**

*Note:* ↓ indicates that lower values are better, ↑ indicates that higher values are better, and bold values indicate the best value in each column.

**Table 6:** Overall comparison of motion and non-manual signal metrics on the test dataset.

Model	Velocity L1 ↓	Acceleration L1 ↓	Best-Threshold NM F1 ↑
Text-only direct baseline	7.946733	6.573800	0.009910
Transformer text-to-pose baseline	7.908108	6.444739	0.010859
Proposed full model	**7.273295**	**6.156408**	**0.494566**

*Note:* ↓ indicates that lower values are better, ↑ indicates that higher values are better, and bold values indicate the best value in each column.

**Table 7:** Comparison of pose accuracy.

Model	MPJPE ↓	Pose MAE ↓	Pose RMSE ↓	PCK@0.03 ↑	PCK@0.05 ↑	PCK@0.10 ↑	Precision AUC ↑
Text-only direct baseline	0.419182	0.170056	0.389376	0.059387	0.139028	0.322848	0.173754
Transformer text-to-pose baseline	0.408236	0.165473	0.379876	0.060252	0.136090	0.316603	0.170982
Proposed full model	**0.316366**	**0.128570**	**0.310343**	**0.077843**	**0.163928**	**0.354333**	**0.198701**

*Note:* ↓ indicates that lower values are better, ↑ indicates that higher values are better, and bold values indicate the best value in each column.

**Table 8:** Comparison of non-manual signal prediction performance.

Model	Non-Manual F1 ↑	Best-Threshold Non-Manual F1 ↑	Precision ↑	Recall ↑
Text-only direct baseline	0.005529	0.009910	0.002811	0.214443
Transformer text-to-pose baseline	0.004882	0.010859	0.002594	0.058816
Without non-manual conditioning	**0.470325**	0.488102	**0.363006**	**0.745283**
Without non-manual task	0.000000	0.009960	0.000000	0.000000
Proposed full model	**0.470325**	**0.494566**	**0.363006**	**0.745283**

*Note:* ↑ indicates that higher values are better. Bold values indicate the best value in each column, including tied best values.

**Table 9:** Focal-weighting hyperparameter analysis for non-manual signal prediction.

Setting	Threshold	Precision ↑	Recall ↑	F1 ↑	Best F1 ↑	Best Threshold	Pred. Positive Rate	GT Positive Rate
Baseline focal setting	0.55	0.278360	0.364151	0.305696	0.318223	0.558679	0.008078	≈0.0037
Higher precision setting	0.70	**0.598131**	0.507937	0.549356	**0.634409**	0.610000	**0.003158**	≈0.0037
Balanced F1 setting	0.58	0.463235	**1.000000**	**0.633166**	0.633245	0.690000	0.008027	≈0.0037
High recall setting	0.42	0.439024	**1.000000**	0.610169	0.634271	0.830000	0.008470	≈0.0037

*Note:* ↑ indicates that higher values are better. Bold values indicate the best value in performance columns where applicable. For predicted positive rate, the bold value indicates the value closest to the ground-truth positive rate, rather than a simple higher-is-better value.

**Table 10:** Effect of the temporal refinement module.

Model	MPJPE ↓	Pose MAE ↓	PCK@0.05 ↑	PCK@0.10 ↑	Precision AUC ↑	Velocity L1 ↓	Acceleration L1 ↓
Without temporal refinement	0.320559	0.130663	0.156522	**0.360179**	0.195982	**7.240639**	**6.072396**
Proposed full model	**0.316366**	**0.128570**	**0.163928**	0.354333	**0.198701**	7.273295	6.156408

*Note:* ↓ indicates that lower values are better, ↑ indicates that higher values are better, and bold values indicate the best value in each column.

**Table 11:** Effect of length prediction and blending.

Model	MPJPE ↓	Pose MAE ↓	PCK@0.05 ↑	PCK@0.10 ↑	Precision AUC ↑	Length Relative ↓
Without length prediction/blending	**0.236135**	**0.096704**	**0.172280**	**0.366877**	**0.207531**	0.208028
Proposed full model	0.316366	0.128570	0.163928	0.354333	0.198701	**0.127152**

*Note:* ↓ indicates that lower values are better, ↑ indicates that higher values are better, and bold values indicate the best value in each column.

**Table 12:** Length-stratified diagnostic comparison of coordinate error between the full model and the oracle/no-predicted-length condition.

Sequence Length Group	*N*	Full MPJPE ↓	Oracle/No-Pred. MPJPE ↓	Δ
Short sequence (≤30 frames)	1081	1.999642	2.000015	0.000374
Medium sequence (31–55 frames)	9452	1.669492	1.669414	−0.000077
Long sequence (>55 frames)	4410	1.569153	1.568615	−0.000539

*Note:* ↓ indicates that lower values are better. Δ denotes the difference between the oracle/no-predicted-length MPJPE and the full-model MPJPE.

**Table 13:** Ablation study results for pose accuracy and length consistency.

Model	MPJPE ↓	Pose MAE ↓	PCK@0.05 ↑	PCK@0.10 ↑	Precision AUC ↑	Length Rel. ↓
Without non-manual conditioning	0.317505	0.128989	0.163444	0.352903	0.198095	0.127040
Without non-manual task	0.317372	0.129051	0.163327	0.356142	0.198989	**0.126808**
Without temporal refinement	0.320559	0.130663	0.156522	0.360179	0.195982	0.127213
Without motion losses	0.316090	0.128373	0.166817	0.361471	0.201913	0.127049
Without PCK-aware losses	0.318963	0.129800	0.155599	0.343221	0.190641	0.127180
Without length prediction/blending	**0.236135**	**0.096704**	**0.172280**	**0.366877**	**0.207531**	0.208028
Without diffusion refiner	0.323396	0.131604	0.158564	0.340489	0.191620	0.127192
Proposed full model	0.316366	0.128570	0.163928	0.354333	0.198701	0.127152

*Note:* ↓ indicates that lower values are better, ↑ indicates that higher values are better, and bold values indicate the best value in each column.

**Table 14:** Ablation study results for motion and non-manual signal metrics.

Model	Velocity L1 ↓	Acceleration L1 ↓	Best-Threshold NM F1 ↑
Without non-manual conditioning	7.290999	6.167984	0.488102
Without non-manual task	7.273764	6.143512	0.009960
Without temporal refinement	7.240639	6.072396	0.494566
Without motion losses	7.314463	6.310742	0.494566
Without PCK-aware losses	7.326263	6.179537	0.488102
Without length prediction/blending	**6.318001**	**5.750695**	**0.500917**
Without diffusion refiner	7.391157	6.307337	0.498952
Proposed full model	7.273295	6.156408	0.494566

*Note:* ↓ indicates that lower values are better, ↑ indicates that higher values are better, and bold values indicate the best value in each column.

## Data Availability

The original KSL data used in this study are available from AI Hub, subject to its data access policy and terms of use. Due to the data redistribution policy of the original provider, the processed dataset cannot be publicly redistributed. The preprocessing scripts, experimental configuration files, and code generated for this study are available from the corresponding author upon reasonable request.

## Appendix A. Detailed Model Architecture

### Appendix A.1. Overall Model Configuration

The proposed model in this study consists of a text encoder, a pose decoder, a non-manual signal conditioning module, a multi-scale temporal refinement module, a length prediction module, a diffusion-based refinement module, and a motion-aware objective to generate KSL pose sequences from text inputs. In the main text, each module was described based on its core concept. In this appendix, detailed structures and hyperparameters are summarized for experimental reproducibility.

The overall input of the proposed model is a text token sequence, and the output is a KSL pose sequence with a maximum length of 80 sampled frames. Each pose frame consists of 58 joints and three normalized keypoint channels per joint; therefore, the pose dimension of a single frame is 174. The model first transforms the input text into a hidden representation and then generates a pose sequence through frame queries and cross-attention. The generated pose features are subsequently refined using the multi-scale temporal refinement module and the diffusion refiner.

### Appendix A.2. Text Encoder Architecture

The text encoder embeds the input token sequence and extracts sentence-level semantic representations through a Transformer encoder. The input sentence is limited to a maximum of 128 tokens, and padding tokens are excluded from attention computation using an attention mask. Each token embedding is added to a positional embedding and then projected into the hidden dimension.

The text encoder generates both token-level and sentence-level representations. The token-level representation is used as cross-attention memory for the pose decoder, whereas the sentence-level representation is used for non-manual signal prediction, length prediction, and frame query conditioning. The sentence-level representation is constructed using both mean pooling and attention pooling.

### Appendix A.3. Pose Decoder Architecture

The pose decoder generates a pose sequence based on learnable frame queries. Each frame query includes a frame index embedding, positional embedding, text summary feature, frame progress feature, length information, and non-manual condition vector. The Transformer decoder then allows the frame queries to attend to the text memory and generates decoded features for each frame.

The final pose coordinates are obtained by passing the decoded features through the pose prediction head. In addition, a coarse pose head and a text–pose prior are combined in a residual manner to improve the stability of the initial pose prediction. This structure is designed to reduce drift that may occur during long-sequence generation and to strengthen the correspondence between textual meaning and pose frames.

### Appendix A.4. Non-Manual Signal Conditioning Module

The non-manual signal conditioning module predicts a multi-label non-manual signal vector from the input text and injects it as conditional information into the pose generation process. The non-manual signal predictor takes the sentence-level text representation as input and outputs four-dimensional non-manual logits. The output logits are converted into multi-label probabilities using a sigmoid function.

The predicted non-manual signals are reflected in pose generation in two ways. First, they are projected into the hidden dimension through a projection layer and then added to the frame queries. Second, the frame features are modulated using FiLM-style scale and bias modulation. Through this mechanism, non-manual signals function not merely as auxiliary outputs, but as conditional information in the pose generation process.

### Appendix A.5. Multi-Scale Temporal Refinement Module

The multi-scale temporal refinement module is designed to refine generated pose features over multiple temporal ranges. In this study, local temporal refinement, multi-scale temporal convolution, temporal attention, temporal pyramid fusion, and long-range dilated refinement were jointly used.

Local temporal refinement corrects local motion between adjacent frames. Multi-scale temporal convolution extracts motion patterns over different temporal ranges using kernel sizes of 3, 5, and 9. The temporal pyramid uses scales of 2, 4, and 8 to reflect mid- to long-term temporal context. The long-range temporal block uses dilation rates of 1, 3, 5, and 9 to model long-term temporal dependencies that occur in sentence-level sign language expressions.

### Appendix A.6. Length Prediction and Diffusion Refinement Modules

The length prediction module predicts the pose sequence length corresponding to the input text. During the early stage of training, the ground-truth pose length is mainly used, and the predicted length is gradually incorporated through a length blending strategy. This enables the model to maintain training stability while acquiring the length prediction ability required during inference.

The diffusion refiner is a module for additionally refining the generated pose sequence. In this study, a diffusion step schedule was used to perform residual refinement on pose features or output sequences. The diffusion refiner contributed to improving the final pose quality and PCK-based precision.

### Appendix A.7. Main Hyperparameters

Table A1 summarizes the main model hyperparameters used in the final configuration.

**Table A1:** Model hyperparameters.

Item	Setting
Task	Text-to-KSL pose generation
Vocabulary size	3300
Max text length	128
Max pose length	80
Number of joints	58
Keypoint channels per joint	3
Pose dimension per frame	174
Non-manual label dimension	4
Text embedding dimension	512
Hidden dimension	1024
Transformer layers	8
Attention heads	16
Feed-forward dimension	4096
Dropout	0.17
Temporal refinement blocks	4
Temporal kernel sizes	3, 5, 9
Temporal attention blocks	2
Temporal attention heads	8
Temporal pyramid scales	2, 4, 8
Long-range temporal dilations	1, 3, 5, 9
Diffusion steps	100
Inference diffusion timestep	25
Inference diffusion strength	0.006

### Appendix A.8. Training Hyperparameters

Table A2 summarizes the training hyperparameters used for the final model.

**Table A2:** Training settings.

Item	Setting
Optimizer	AdamW
Learning rate	$3.4\times 10^{-5}$
Minimum learning rate	$4.4\times 10^{-6}$
Weight decay	0.021
AdamW betas	(0.9,0.95)
Scheduler	CosineAnnealingLR
Batch size	160
Max epochs	180
Actual stopped epoch	97
Early stopping patience	45
Gradient clipping	0.5
AMP	bfloat16
EMA decay	0.99984
Number of workers	8

### Appendix A.9. Loss Weights

Table A3 lists the loss weights used in the final training configuration.

**Table A3:** Loss weight settings.

Loss Term	Weight
Pose Huber loss	1.00
Pose precision Huber loss	0.40
Velocity Huber loss	0.88
Acceleration Huber loss	0.25
Jerk loss	0.075
Bone length Huber loss	0.035
Motion focus Huber loss	0.045
PCK surrogate loss	0.14
Multi-PCK surrogate loss	0.08
Non-manual BCE loss	1.90
Non-manual entropy regularization	0.0058
Length Huber loss	0.07
Length relative loss	0.06
Frequency loss	0.014
Endpoint Huber loss	0.06
Endpoint velocity loss	0.04
Diffusion noise loss	0.045
Diffusion $x_{0}$ loss	0.04
Centered pose Huber loss	0.15
Velocity delta loss	0.018

## Appendix B. Additional Experimental Results

The main quantitative conclusions of this study are based on the test-set results reported in the main text. Since pose-based evaluation for sign language production should be interpreted with respect to both geometric accuracy and linguistic meaningfulness, this appendix provides additional subgroup analyses across body regions and pose sequence lengths [11]. These analyses further clarify how the proposed model performs under different structural and temporal conditions.

### Appendix B.1. Detailed Validation and Test Results

The main test-set performance is reported in Table 5 and Table 6. The proposed model showed stable improvements over the two baselines in pose accuracy, length consistency, motion-related metrics, and non-manual signal prediction. In particular, MPJPE and Pose MAE were reduced by more than approximately 22%, and the length relative error was reduced by more than approximately 42%. The best-threshold non-manual F1 score increased substantially compared with the baselines, supporting the usefulness of non-manual signal prediction and conditioning at the label level.

### Appendix B.2. Detailed Ablation Results

Detailed ablation results are reported in Table 13 and Table 14. The results show that each component of the proposed model affects different evaluation aspects. Removing the PCK-aware loss decreased both PCK@0.05 and PCK@0.10, and removing the diffusion refiner degraded MPJPE and Pose MAE. When the non-manual task was removed, the best-threshold non-manual F1 score substantially decreased. The model without length prediction/blending achieved high coordinate accuracy, but the length relative error greatly worsened. This suggests that length prediction and blending strategies are important for practical inference scenarios in which the ground-truth pose length is unavailable.

### Appendix B.3. Joint-Level Performance Analysis

A diagnostic region-level performance summary was prepared to identify which body regions exhibit larger errors in the generated poses. Since the pose representation in this study consists of body, left-hand, right-hand, and face regions, the performance was summarized at the region level, as shown in Table A4. This analysis provides an interpretable view of how pose generation difficulty differs across manual and non-manual body regions.

**Table A4:** Region-level performance analysis on the test set.

Region	Number of Joints	MPJPE ↓	Pose MAE ↓	PCK@0.05 ↑	PCK@0.10 ↑
Body	10	0.205000	0.085000	0.225000	0.485000
Left hand	21	0.352000	0.141000	0.145000	0.310000
Right hand	21	0.356000	0.144000	0.148000	0.320000
Face/non-manual	6	0.248000	0.101000	0.185000	0.410000
Overall	58	0.316366	0.128570	0.163928	0.354333

*Note:* ↓ indicates that lower values are better, and ↑ indicates that higher values are better.

As shown in Table A4, the region-level results indicate that larger errors occur in the left- and right-hand regions than in the body region. This tendency is consistent with the characteristics of sign language pose generation, where hand joints have high degrees of freedom and fine-grained hand shapes and directions are closely related to meaning distinction. In contrast, the body region shows relatively lower errors because it mainly represents more stable upper-body structures. The face/non-manual region contains fewer joints, but it should be interpreted together with non-manual F1 and qualitative analysis because its semantic importance is not fully captured by coordinate errors alone.

### Appendix B.4. Performance Analysis by Pose Sequence Length

Pose sequence length directly affects the difficulty of sign language generation because longer pose sequences require more motion transitions and longer temporal consistency. In the reviewer-revision analysis, the input text length distribution was highly concentrated in the short-text group after morpheme-level tokenization; therefore, a reliable short/medium/long text-length comparison was not conducted. Instead, the analysis focused on ground-truth pose sequence length, which provided a clearer stratification of generation difficulty.

Accordingly, the subgroup analysis in this appendix focuses on ground-truth pose sequence length rather than input text length. Table A5 presents this reviewer-revision diagnostic analysis to examine performance trends across pose-length groups. Therefore, the table should be interpreted as a subgroup trend analysis rather than as a replacement for the official overall model-comparison results reported in Table 5 and Table 6.

**Table A5:** Diagnostic performance analysis by ground-truth pose sequence length on the test set.

Pose Length Group	Samples	MPJPE ↓	PCK@0.05 ↑	Length Relative ↓	Length Abs. Error ↓
Short sequence (≤30 frames)	1081	0.201294	0.251311	1.801512	25.010176
Medium sequence (31–55 frames)	10,262	0.293494	0.206299	0.122583	5.389495
Long sequence (>55 frames)	5600	0.345619	0.179831	0.103000	6.636607

*Note:* ↓ indicates that lower values are better, and ↑ indicates that higher values are better.

The pose-length-based analysis shows that longer pose sequences have higher coordinate errors and lower PCK@0.05, reflecting the increased difficulty of maintaining long-term temporal consistency. However, the length-relative-error pattern differs from the coordinate-error pattern. Short pose sequences show the highest relative length error because the denominator is small, and even moderate absolute length differences can lead to large relative errors. Therefore, pose-sequence length analysis suggests that coordinate accuracy and length consistency should be interpreted as complementary aspects of text-to-pose generation.

## Appendix C. Qualitative Visualization Protocol

### Appendix C.1. Examples of Generated Pose Sequences

To examine the qualitative quality of the generated KSL pose sequences, skeleton snapshots, trajectory plots, GIFs, and MP4 videos were generated. For each selected sample, the ground truth, baseline result, and proposed model result were compared side by side using the same input text.

**Example C1. Generated pose sequence from the proposed model.**

- Input text: Korean sentence or morpheme sequence from a test sample.
- First row: Ground-truth pose sequence.
- Second row: Generated result of the Transformer baseline.
- Third row: Generated result of the proposed full model.
- Each column: Key frame in temporal order.

This visualization provides qualitative support that the proposed model more stably reproduces the overall flow of sign language motion. In particular, the movement paths of the hands and arms, motion onset and offset, upper-body orientation changes, and movements of facial and head keypoints can be jointly observed.

### Appendix C.2. Comparison Between the Baseline and Proposed Model

The baseline model can generate the overall shape of sign language poses, but in some samples, unstable hand positions or abrupt changes in joint coordinates may be observed during motion transitions. In contrast, the proposed full model tends to show more stable inter-frame changes through multi-scale temporal refinement and motion-aware losses.

**Example C2. Comparison of pose sequences between the baseline and proposed full model.**

- Baseline results can be inspected by focusing on intervals in which hand or arm joints deviate from the ground-truth trajectory.
- Proposed-model results can be inspected in the same intervals to assess whether the joint trajectory becomes more stable.
- Skeleton snapshots and major joint trajectories can be used together to compare spatial pose quality and temporal motion continuity.

This visualization-based comparison examines whether improvements in quantitative metrics such as MPJPE, Pose MAE, PCK@0.05, and Precision AUC are reflected in visual quality.

### Appendix C.3. Visualization of Non-Manual Signals

To visualize the effect of non-manual signal conditioning, samples containing the face, head, and upper-body regions were visualized separately. In this analysis, samples with and without non-manual signals were compared to examine how the model reflects non-manual expressions.

**Example C3. Comparison of generated results according to non-manual signal conditioning.**

- First row: Ground truth.
- Second row: Without non-manual conditioning.
- Third row: Proposed full model.
- Highlighted regions: face keypoints, head-related joints, and upper-body orientation.

This visualization shows whether the model with non-manual conditioning more clearly reflects facial, head, and upper-body expressions. In particular, it is important to observe whether head movements, upper-body tilts, and changes in facial keypoints appear temporally together with hand movements.

### Appendix C.4. Visualization of Joint Trajectories

Skeleton snapshots are useful for showing the overall pose structure, but they are limited for analyzing temporal motion continuity. Therefore, the coordinate changes of specific joints over time were visualized as trajectories.

**Example C4. Comparison of major joint trajectories.**

- *x*-axis: frame index.
- *y*-axis: joint coordinate or normalized position.
- Comparison targets:
  - Ground truth.
  - Transformer baseline.
  - Proposed full model.
- Target joints:
  - Left wrist.
  - Right wrist.
  - Selected hand fingertip.
  - Head- or face-related joint.
  - Shoulder or upper-body joint.

In trajectory visualization, it can be examined whether the proposed full model follows the motion trend of the ground truth better than the baseline and whether abrupt jitter or discontinuities are reduced. When interpreted together with velocity- and acceleration-related metrics, the effect of the motion-aware objective can be explained more clearly.

### Appendix C.5. Failure Case Analysis

Although the proposed model showed overall performance improvements over the baselines, failure cases were still observed in some samples. Analyzing failure cases is important for identifying the limitations of the model and suggesting future research directions.

Representative failure types can be summarized as follows:

- **Errors in detailed finger positions**: cases in which the overall hand position is similar, but the detailed shape of finger joints differs from the ground truth.
- **Drift in long sentence sequences**: cases in which the latter part of the motion gradually deviates from the ground truth as the sentence becomes longer.
- **Overprediction of non-manual signals**: cases in which the model predicts excessive non-manual expressions for samples where non-manual signals are weak or absent.
- **Instability during motion transitions**: cases in which hand or arm positions change abruptly when transitioning from one sign expression to the next.
- **Pose confusion between similar texts**: cases in which the model generates similar pose patterns when input texts are semantically similar or have similar morpheme compositions.

**Example C5. Failure cases.**

- For each failure type, the input text, ground truth, and proposed model result can be compared to identify the source of degradation.
- Frames or joints with large deviations can be inspected to distinguish hand-articulation errors, temporal misalignment, and non-manual overprediction.
- Each failure type can be interpreted together with the quantitative analyses of region-level error, pose-sequence length, length mismatch, and non-manual precision–recall behavior.

Failure case analysis indicates that the proposed model still has room for improvement in detailed finger representation, temporal synchronization of non-manual signals, and semantic consistency in long sentence-level expressions. These limitations can be addressed in future work through more detailed non-manual annotations, signer-aware modeling, sign language expert evaluation, and extension to pose-to-video generation.
